# Long‐Term Care Partnership Effects on Medicaid and Private Insurance

**DOI:** 10.1002/hec.4949

**Published:** 2025-03-15

**Authors:** Joan Costa‐Font, Nilesh Raut

**Affiliations:** ^1^ London School of Economics and Political Science London UK; ^2^ IZA, Bonn & CESIfo Munich Germany

**Keywords:** difference‐in‐differences, long‐term care insurance, long‐term care partnerships, Medicaid, United States

## Abstract

We examine the impact of the Long‐Term Care Insurance Partnership (LTCIP) program—a collaborative initiative between the state‐level Medicaid programs and private health insurance companies designed to promote private long‐term care insurance (LTCI)—on insurance ownership and Medicaid utilization. We draw on individual‐level longitudinal data and employ a difference‐in‐differences (DD) design adjusted for the staggered implementation of the program between 2005 and 2018. Our results suggest that the rollout of the LTCIP program led to a 1.54 percentage point (pp) (14.7%) increase in LTCI ownership and a 0.82 pp (13.3%) reduction in Medicaid uptake. Our estimates suggest that these combined effects led to an approximate average cost saving of $74 per 65‐year‐old participant. These findings are explained by a certain degree of substitution between LTCIP and traditional LTCI contracts, ultimately postponing the use of Medicaid benefits.

## 1 Introduction

Changes in the eligibility criteria for insurance covering long‐term care services and supports (LTCSS) can have profound financial implications for both households and the financial sustainability of last resort public insurance programs such as Medicaid. Two‐thirds of Americans aged 65 and above are expected to use LTCSS at some point in their life (Congressional Budget Office 2013; Eggleston and Fuchs 2012; Eggleston and Mukherjee 2019; Kemper, Komisar, and Alecxih 2005). However, LTCSS are not affordable for many Americans. For instance, in 2017, the average monthly costs of LTCSS in a nursing home stood at $8385 (AALTCI 2019; CMS 2018),^1^ which is about four times the average monthly income of individuals over 65 in the country. To date, it is unclear how best to fund LTCSS in a country like the United States (US), and especially what role a long‐term care insurance (LTCI) should play.

Public insurance programs already fund 72% of spending on LTCSS in the US. Medicaid—a public insurance program for low‐income families jointly funded by state and federal governments—alone accounts for approximately 53% of total LTCSS expenditures. The remaining 19% is covered by other public programs, including Medicare, the Veterans Health Administration, State CHIP, general assistance programs, and various state and local initiatives (AARP 2019; Kaiser Family Foundation 2019; Reaves and Musumeci 2015; Thach and Wiener 2018). Only the remaining 28% of LTCSS spending is privately financed respectively by LTCI ownership (11%) and out‐of‐pocket expenses (17%) (Thach and Wiener 2018; Reaves and Musumeci 2015). However, the lower rate of LTCI ownership can exacerbate both the financial and care burden of families and place additional strain on Medicaid, as private insurance benefits are typically required to be almost depleted before individuals qualify for Medicaid‐funded care (Pauly 1990; Brown and Finkelstein 2008; Goda 2011).

In a country with limited appetite for further federal public insurance expansion, the expansion of LTCI ownership can give rise to substantial cost savings by covering long‐term care services (LTCSS) that would otherwise be ineficiently funded out of personal savings. Without LTCI, individuals are either more likely to incur in significant out‐of‐pocket costs when using LTCSS, or depend on means tested Medicaid funding (Brown and Finkelstein 2008; Finkelstein and McGarry 2006; Goda 2011). However, barely 11% of individuals over the age of 50 report having purchased LTCI in the Health and Retirement Survey (HRS). This paper examines a relevant policy question, namely how best to encourage the purchase of LTCI. Encouraging the purchase of LTCI is important given that private insurance is required to pay first, even when individual meets both the Medicaid income and asset means‐testing requirements. Hence, expanding LTCI ownership can help the long‐term sustainability of Medicaid.^2^

The expansion of LTCI faces both demand and supply side challenges (E. Norton 2016).^3^ Such challenges include the limited perceived value of the insurance contracts *when insurers limit the benefits and rise the premiums over time*, as well as, the distrust with insurance companies and the presence of adverse selection, namely the risk of high risk individuals selecting into purchasing LTCI (Oster et al. 2010).^4^ Finally, from a supply side, insurers tend to exit the market when it is perceived as highly uncertain, and when they stay, they engage in underwriting or use indemnity contracts that put a cap of potential loses (Cutler 1996; Konetzka 2014). Finally, the slow penetration of LTCI can result from individuals strategic spend down of their wealth to qualify for Medicaid, which in turn poses significant challenges to both state and federal budgets (Pauly 1990; Brown, Goda, and McGarry 2012; Goda, Golberstein, and Grabowski 2011; Mommaerts 2024).

To address some of the above‐mentioned constraints, several U.S. states have adopted the so‐called Long‐Term Care Insurance Partnership (LTCIP) program (Meiners and Goss 1994). The LTCIP program was initially intended to contain the expansion of Medicaid uptake by stimulating LTCI ownership.^5^ A key feature of the LTCIP program is that it allows individuals to retain assets up to the value of their LTCI coverage and still qualify for Medicaid, if they meet other eligibility requirements.^6^ To date, there are only a handful of early studies which attempted to estimate impact of LTCIP on LTCI ownership. All of them refer to its early introduction and document no statistically significant impact of LTCIP on LTCI ownership (Pan 2009; Lin and Prince 2013; Bergquist, Costa‐Font, and Swartz 2018). However, the effect of LTCIP may take some years to exert any effect, especially on Medicaid uptake (Robert Wood Johnson Foundation (RWJF), 2007; Pan 2009; Lin and Prince 2013; Bergquist, Costa‐Font, and Swartz 2018). However, previous studies have not examined the effects of the program expansion (see Table 1) after the deficit reduction act (DRA)^7^ in 2005 normalized the LTCIP program in multiple U.S. states.^8^

**TABLE 1:** The US state adoption of long‐term care insurance partnership (LTCIP) program.

Post DRA‐2005 partnership adoption	States	Inclusion/Exclusion status	Quantity
Treated states in 2006	ID, MN, NE	Included as **partnership state** in 2006	3
Treated states: 2007–2008	AZ, AR, CO, FL, GA, KS, KY, MO, ND, NJ, NV, OH, OK, OR, PA, RI, SD, TN, TX, VA	Included as **partnership states** in 2008	20
Treated state: 2009–2010	AL, IA, LA, ME, MD, MT, NH, SC, WI, WY	Included as **partnership states** in 2010	10
Treated states: 2011–2012	DE, NC, WA, WV	Included as **partnership states** in 2012	4
Never‐ treated states	AK, DC, HI, IL, MA, MI, MS, NM, UT, VT	Included as **non‐partnership** states	10
Permanent partnership states (always treated: From 1994)	CT, CA, NY, IN	One of the control variables	4

*Note:* The Deficit Reduction Act (2005) came in force in February 2006. State‐wise (50 states + DC) information on the adoption of the LTCIP program is obtained from American Association of Long‐Term Care Insurance website, which comes under U.S. Government Accountability Office's Consumer Information Center. Refer Table S1 of the Appendix for more details. The permanent partnership states are states which always had partnership program because these four stated had partnership policy in place from 1994.

This paper examines whether the gradual adoption of the LTCIP program by US states gave rise to both an increase in the purchase of LTCI and Medicaid enrollment. We use a Difference‐in‐Differences (DiD) design to identify the effect of the rollout of LTCIP across states, while also exploring different specifications that account for the staggered implementation of the program. We draw on a comprehensive longitudinal dataset that follows individuals for more than two decades (1996–2018). Next, to better understand the characteristics of individuals taking up LTCIP policies, we examine the heterogeneous effects across household composition, and we carry out a series of robustness checks, placebo tests as well as an analysis of the short‐term effects of the rollout of LTCIP programs which in turn replicates the findings of earlier studies (Lin and Prince 2013). Finally, the paper provides a welfare evaluation of the impact of the LTCIP program.

We make four contributions to the literature. First, earlier contributions examining the effect of LTCIP programs were confined to the period leading up to 2008, hence focusing on the immediate impact on LTCI ownership (Lin and Prince 2013). However, LTCIP's exhibited a significant expansion post‐2005, coinciding with the enrollment of numerous states into the program.^9^ Hence, considering a longer time frame allows for a more precise identification of the effect.

Second, we examine the full temporal scope of the LTCIP adoption by examining the effect until 2018 before the COVID‐19 pandemic. Third, unlike previous research, we examine the effect of the adoption of LTCIP's on Medicaid uptake, which might help reducing the problem of the implicit tax that Medicaid poses on LTCI as discussed in Section 2.1. Fourth, we extend our study with a simulation analysis to estimate average Medicaid savings per beneficiary (Goda 2011). Lastly, we empirically document evidence that allows identifying some of the mechanisms driving the effect, and more specifically the role of individuals health, income and wealth as well as household composition.

The next section describes the relevant institutional background explaining how long‐term care is funded in the U.S. and the LTCIP program. Next, we report a description of the data and the empirical strategy used. Section four displays the results, section five provides robustness checks, and a final section concludes the paper.

## 2 Institutional Background

### 2.1 Funding Long‐Term Care in the United States

The funding of LTCSS in the US is based on a combination of public and private insurance schemes. As mentioned, more than half of LTCSS is funded by Medicaid, a means‐tested program that is jointly financed by state and federal governments (Reaves and Musumeci 2015, AARP 2019; Kaiser Family Foundation 2019; Thach and Wiener 2018).^10^ In contrast, Medicare only provides short‐term stay coverage in a skilled nursing home (AARP 2019). However, Medicaid means‐testing eligibility constraints expose all but the poorest individuals to the financial risk of bearing considerable amount of out‐of‐pocket expenses (Brown and Finkelstein 2008). Simultaneously, the limited Medicaid coverage depresses the demand for insurance as it imposes an implicit tax on LTCI (Norton and Sloan 1997; Brown and Finkelstein 2008, 2011). Hence, an important question to examine is whether it is possible to improve the design of Medicaid so that it does not crowd out the purchase of LTCI. That is precisely the main purpose of the partnership program (LTCIP). If the LTCIP attains its intended goals, it could incentivize LTCI ownership and at the same time delay the uptake of Medicaid. Hence, it can, help contain the rise in Medicaid spending.

### 2.2 The Long‐Term Care Insurance Partnership (LTCIP) Program

The LTCIP program refers to an intervention meant to incentivize LTCI ownership and constrain Medicaid spending (Robert Wood Johnson Foundation (RWJF) 2007). That is, the purpose of the program was to encourage the purchase of LTCI among middle‐income individuals who otherwise would neither purchase LTCI nor qualify for Medicaid.^11^, ^12^

The LTCIP enables policyholders to exclude their long‐term care expenses, typically up to the amount covered under their individual LTCIP contract coverage, when qualifying for Medicaid means tests (the model is also known as the ‘dollar‐for‐dollar’).^13^ That is, a LTCIP contract allows individuals who deplete their LTCI coverage to safeguard assets equal to the insurance benefits paid, without having to spend down to Medicaid's strict asset limits. For example, an insurance policy for a 65‐year‐old individual, with a median wealth of $144,000, provides a daily benefit of $100 per day for two years, thus an individual can protect an asset worth of $73,000 (=365 × 100 × 2) (Brown and Finkelstein 2011). Therefore, in a state that has a $2000 income eligibility threshold, she needs to spend down the remaining $69,000 worth of assets (resulting from $144,000–$73,000–$2000) to become eligible for Medicaid financed care. The LTCIP program nevertheless offers an incentive to both protect individuals' assets and at the same time reduce their future Medicaid spending by stimulating the coverage of LTCI (Rothstein 2007; Bergquist, Costa‐Font, and Swartz 2018).

The LTCIP program was initially introduced in 1987 through the efforts of the Robert Wood Johnson Foundation (RWJF), as ‘The Federal Long‐Term Care Insurance Program’, and it was piloted in four states—commonly referred to as RWJF states—, namely California (1994), Connecticut (1992), Indiana (1993), and New York (1993) Alper (2006). After a federal legislation moratorium, no LTCIP programs were established for more than 20 years. In this paper, we call such four initial designs in four states as ‘permanent partnership states’ (see Table 1), and we control for them as they were large experimental states. However, this paper focus is on the new programs developed after the moratorium was lifted in 2006 as part of the implementation of the federal Deficit Reduction Act of 2005 (DRA‐2005). Table 1 displays the year‐wise adoption of LTCIP programs by U.S. states. Since 2006, numerous states have gradually adopted the LTCIP program, which unlike the permanent partnership schemes, have been standardized its provisions and are comparable across states, creating a quasi‐experimental set‐up that can be exploited to identify the causal effect of the introduction of a LTCIP in a state.

### 2.3 Interaction of the Partnership Program and Medicaid

A key feature of the LTCIP design is its dollar‐for‐dollar asset protection for insurance purchasers. Without the LTCIP program, a typical individual would either need to spend down their assets to qualify for Medicaid or purchase LTCI to cover their long‐term care expenses. Meiners (2009) refers to such individual in the middle of the income and asset distribution as the Middle‐Middle (MM) resource group—an individual with considerable savings and monthly income that can ensure a comfortable life in the absence of long‐term care needs.

The MM group is made of individuals with monthly income in the range of $1000 ‐ $5000 and total assets between $100,000 and $350,000 (GAO 2007). If such group of individuals over‐insure their assets by purchasing a LTCIP policy, then they may end up reducing their Medicaid‐related costs. In contrast, in the absence of a LTCIP, MM individuals would typically underinsure their assets, as they might struggle to afford adequate coverage, and would be more likely to deplete their assets to qualify for Medicaid if they require LTCSS. However, the dollar‐for‐dollar asset protection provided by the LTCIP allows those with substantial wealth to finance their care through insurance, enabling them at the same time to purchase coverage beyond what is necessary to protect their assets (Meiners 2009). Nevertheless, it is an empirical question whether the additional coverage provided by the LTCIP managed to reduce Medicaid costs, potentially leading to Medicaid savings.

Finally, it's worth mentioning that an individual's behaviour can be altered by the Medicaid estate recovery rules, which is designed to recover Medicaid costs from an individual's estate after death. However, when the LTCIP is in place in a state, individuals who purchase LTCI can protect an amount of assets equal to the benefits paid by their policy. Hence, such asset protection is typically excluded from Medicaid's asset eligibility calculations, allowing individuals to qualify for Medicaid while retaining a greater portion of their assets, which are not subject to Medicaid estate recovery^14^ Thus, LTCIP offers a safety net against the combined demands of estate recovery and Medicaid eligibility.

### 2.4 Expected Effects of the State Adoption of LTCIP Program

We expect LTCI ownership to rise in states implementing LTCIP, particularly among individuals with some assets to protect. The take up of the LTCIP program should be more  pronounced among middle‐income individuals who might otherwise expect to spend down their wealth to be eligible for Medicaid—those who are neither wealthy enough to self‐insure nor poor enough to expect to qualify for Medicaid.

Another relevant effect of the LTCIP program is that it simplifies Medicaid planning for individuals. Indeed, Medicaid planning in non‐LTCIP states is more complex and lacks strong incentives for effective planning. As a result, Medicaid planning becomes a key mechanism through which LTCIP influences LTCI ownership. That is, as more individuals purchase LTCI through the LTCIP program, they are expected to delay or avoid Medicaid reliance by using LTCI to cover long‐term care expenses. Hence, they may only seek Medicaid when the LTCI benefits are exhausted while retaining some of the assets covered by the LTCIP policy. This gives rise to a reduced Medicaid uptake in the short term as private LTCI provides pay for LTCSS. In the long run if individuals need care beyond their LTCIP coverage, Medicaid uptake may still occur but it would only be for a smaller share of eligible individuals. The remainder of this paper aims at empirically testing these effects.

## 3 Data and Empirical Strategy

### 3.1 The Data

We use the Health and Retirement Study (HRS), a large‐scale longitudinal dataset sponsored by the National Institute of Aging (NIH). The HRS is a biennial survey that began interviewing respondents and their spouses from 1992 onward. The first wave of HRS collected information from individuals aged 50 and above (mainly aged 51–61 and born between 1931 and 1941) when the sample was first collected in 1992 (National Institute on Aging and The Social Security Administration 2018). The HRS contains an even older cohort, that is, people born before 1923, named Asset and Health Dynamics among the Oldest Old (AHEAD). Starting in 1993, the AHEAD sample was collected every alternate year until 1998 when it was merged with the main sample. Subsequently, two additional sample cohorts were added, namely the War Baby (WB ‐ Individuals born between 1942 and 1947) and the Children of Depression Age (CODA ‐ Individuals born between 1924 and 1930) cohorts.^15^

We draw on restricted HRS data from 1992 through 2018, which allows us to identify state information to locate the state residence for all sampled individuals. However, we remove the first two waves (1992 and 1994) from our main sample due to the vagueness in the questions' wording. An extensive study by AHIP (2007) reports that close to 90% of LTCI buyers fall under the age of 75, indicating that the vast majority of LTCI purchasers take place before reaching such age, with the average age of LTCI buyers in this survey is between 61 and 62. Thus, we limit our analysis to individuals aged 50–75.^16^ The final sample consists of data from 1996 through 2018 which has 156,102 observations and 32,329 sample individuals.

Next, we matched the final sample with the detailed information of the LTCIP policy implementation for each state at each time period *t*. Specifically, we identify whether a state adopted the LTCIP program in each time *t*. The policy variable is coded as 1 if an individual resides in a state that implemented a LTCIP program, and 0 otherwise. This approach enables us to compare individuals in states that offer a LTCIP contracts (defined as “partnership states”) to those that do not (defined as “non‐partnership states”). A detailed description of all variables is provided in Table S7 of the Appendix.

The HRS sampling is based on a multi‐stage area probability design that includes geographical clustering, oversampling specific demographic groups, and containing area stratification (Sonnega et al. 2014). Each sampled housing unit is subjected to a quick household screening interview to ascertain eligibility. The age and couple status of each adult living in the home (age 18+) are provided. A primary respondent is chosen at random from among all household members who are of legal age, and if they are married or cohabitating, their spouse or partner is likewise drawn from the sample, regardless of age. Attempts to screen households have been made in 1992, 2004, and 2010. Subject to extra efforts taken by HRS staff for minimizing attrition rate, the HRS has a reasonably high response rate.^17^ Nevertheless, efforts of HRS in bringing back respondents to the sample after non‐participation in one or more interviews have helped mitigate the bias caused by attrition. The analysis presented by the existing literature suggests that this strategy of bringing back temporary non‐respondents substantially helps in reducing selection bias and in mitigating threats to external validity (Kapteyn et al. 2006; Banks, Muriel, and Smith 2011; Michaud et al. 2011).

Finally, it is worth mentioning that in this study, we do make use of sample weights at the individual level and we cluster the standard errors at the state level as the LTCIP program is a state specific program.

### 3.2 Descriptive Evidence

Table 2 displays the descriptive statistics for the sample. We report that approximately 11% of sampled individuals had purchased LTCI in our sample and 8.6% were enrolled in Medicaid. The average individual in the sample is close to 63 years of age. Approximately 75% of sample individuals are White‐Americans and slightly more than a quarter reported being in fair or poor health. Table 3 further describes the sample characteristics based on treatment status. As expected, partnership states exhibit both a higher share of individuals holding LTCI contracts, and exhibit a higher Medicaid uptake compared to never‐treated states. In contrast, both samples are otherwise balanced in terms of other characteristics such as income, wealth, age, gender, marital status, and college education.

**TABLE 2:** Summary statistics of individual level characteristics.

Variables	*N*	Mean	Std dev	Min	Max
LTCI	156,102	0.107	0.31	0	1
Medicaid	156,102	0.086	0.28	0	1
Income (US$)	156,102	70,921	218,754	0	60,000,000
Wealth (US $)	156,102	429,544	1,441,925	−3,624,527	9,987,000
Age	156,102	62.6	6.83	50	75
Male	156,102	0.43	0.50	0	1
Married	156,102	0.66	0.48	0	1
College/More	156,102	0.46	0.50	0	1
Children	153,626	0.93	0.26	0	1
White	156,102	0.74	0.44	0	1
Retired	136,624	0.54	0.50	0	1
Fair/Poor health	156,102	0.26	0.44	0	1
Cancer	156,102	0.11	0.31	0	1
Arthritis	156,102	0.51	0.50	0	1
Diabetes	156,102	0.20	0.40	0	1
Stroke	156,102	0.06	0.23	0	1
Heart disease	156,102	0.18	0.38	0	1
Psychological disease	156,102	0.15	0.36	0	1
Lung disease	156,102	0.08	0.27	0	1

*Note:* This table provides description of the relevant variables used from the Health and Retirement Study, Waves 3–14, year 1996–2018. The present sample is restricted to individuals aged 50–75. The first two variables refer to the ownership of LTCI and take‐up of Medicaid, alongside the health and demographic indicators for individuals in the sample.

**TABLE 3:** Sample characteristics based on partnership (treatment) status.

Variables	Partnership states		Non‐partnership states	
	Mean	Std dev	Mean	Std dev
LTCI	0.11	0.31	0.098	0.30
Medicaid	0.076	0.27	0.084	0.28
Income (US$)	69,282	162,433	68,090	117,792
Wealth (US$)	407,896	1,167,943	405,287	1,121,934
Age	62.7	6.84	62.5	6.8
Male	0.43	0.50	0.43	0.50
Married	0.66	0.47	0.66	0.47
College/More	0.44	0.46	0.46	0.5
Children	0.93	0.25	0.92	0.27
White	0.76	0.42	0.72	0.45
Retired	0.55	0.49	0.55	0.50
Fair/Poor health	0.26	0.44	0.24	0.43
Cancer	0.11	0.31	0.11	0.31
Arthritis	0.51	0.50	0.54	0.50
Diabetes	0.19	0.40	0.19	0.39
Stroke	0.06	0.23	0.06	0.23
Heart disease	0.18	0.39	0.18	0.39
Psychological disease	0.15	0.36	0.16	0.37
Lung disease	0.09	0.28	0.08	0.27

*Note:* This table provides comparison of averages for partnership (treated) versus non‐partnership (never‐treated) states for all the variables in the sample using Health and Retirement Study, Waves 3–14, year 1996–2018.

**FIGURE 1:**
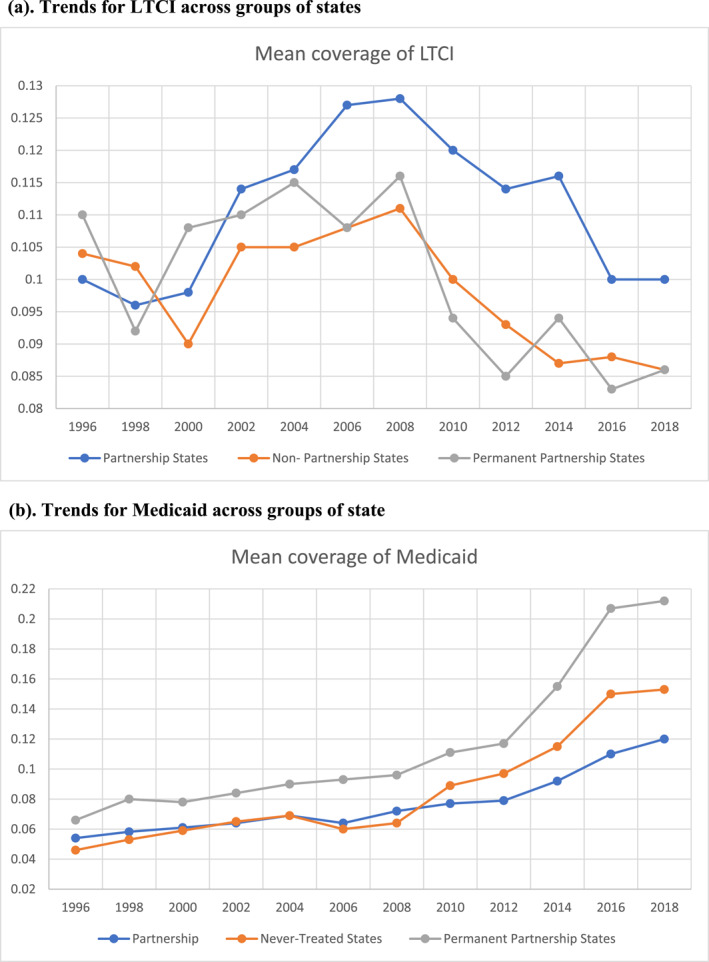
(a) Trends for LTCI across groups of states. That is, it reports the trends in the mean coverage of LTCI for each cohort of states adopting the LTCIP, for partnership, permanent partnerships and for non‐partnership (never‐treated) states using Health and Retirement Study, Wave 3–14, 1996–2018. (b). Trends for Medicaid across the groups of states described in (a). That is, it reports the trends in the mean coverage of Medicaid for each cohort of states adopting the LTCIP program for partnership, permanent partnerships and non‐partnership (never‐treated) states using Health and Retirement Study, Wave 3–14, 1996–2018.

Next, Figure 1a depicts the trends in the proportion of individuals that have purchased LTCI in LTCIP states and non‐LTCIP states (distinguishing between permanent partnership states and never‐treated states). Figure 1a displays, as expected, evidence suggesting that the introduction and subsequent rollout of partnership programs increased LTCI ownership in the treatment states (LTCIP) compared to control states (non‐LTCIP). However, the figure reveals too a sharp decline in LTCI coverage after 2008 which was larger in non‐partnership states, largely attributed to the effect of the financial crisis alongside a significant drop in the total market policy sales after 2002. Figure S3 in the Appendix illustrates that the trends in the sales of insurance policies continued to decline after 2002 through 2014.^18^ The introduction of LTCIP came at a time when sales of LTCI policies were declining, and managed to partially counteract the trend by encouraging further the purchase of LTCI.^19^ Hence, it appears that the descriptive trends are consistent with the idea that the LTCIP program was effective in steering LTCI purchase.^20^

Additionally, Figure 1b displays the trends of Medicaid uptake over time for LTCIP states, and non‐LTCIP states (distinguishing between permanent partnership states and never‐treated states). The pre‐policy trends indicate that Medicaid take‐up in LTCIP states and never treated states were very similar, suggesting evidence consistent with parallel trends. The figure also shows a gradual shift in Medicaid uptake trends after 2008, with as expected indicate that Medicaid take‐up was lower in LTCIP states compared to never‐treated states. Notably, Medicaid uptake in never‐treated states increased after the implementation of LTCIP program. These two figures visually illustrate descriptive evidence of an association between the LTCIP program expansion and both LTCI ownership and Medicaid uptake.

### 3.3 Empirical Strategy

To causally identify the effect of LTCIP, we use a generalized Difference‐in‐Differences (DiD) design to compare the LTCI ownership in Partnership states to that of non‐Partnership states after the introduction of the LTCIP program in a state. Equation (1) below describes our difference‐in‐differences specification, namely a two‐way fixed effects (TWFE hereafter) estimator. In a first instance, we use TWFE specification to obtain our main baseline estimates. However, the recent literature suggests that such estimates can be biased, and some adjustments are needed, when the treatment varies across different time periods for various groups (Borusyak and Jaravel 2017; Callaway and Sant’Anna 2021; de Chaisemartin and D'Haultfoeuille 2020; L. Sun and Abraham 2020; Goodman‐Bacon 2021). To address the potential bias in a TWFE specification, Goodman‐Bacon (2021), suggested a weighted average of all the existing 2x2 DiD estimators. In our specification, given the staggered introduction of LTCIP programs, TWFE estimates can be biased because of both the heterogenous treatment and the staggered treatment timing. Thus, we also estimate the CSDID model using the treatment effect measures for various treatment comparisons (Callaway and Sant’Anna 2021). Our focus is on estimating the effect of treated groups compared to never treated where each cohort of states adopting the LTCIP program in a specific year is compared to never treated states.

We employ a linear probability model or LPM (as well as non‐linear models in the robustness checks section), as the interpretation of the interaction terms is more straightforward (Ai and Norton 2003; S. Athey and Imbens 2006; Puhani 2012). Our dependent variables refer to LTCI ownership and Medicaid uptake over the period from 1996 through 2018. As discussed states implementing the LTCIP program, or partnership states began deploying its programs only after 2005. Hence, our generalized difference‐in‐differences specification is as follows^21^:

(1) $$Y_{\text{ist}\;=}\;\beta_{0}+\beta_{1}{\text{Partnership}}_{\text{st}}{+\rho X}_{\text{ist}}{+\theta }_{s}+\sigma_{t}+\epsilon_{\text{ist}}$$

where *Y*_ist_ refers to either LTCI ownership or Medicaid uptake for an individual (*i*) in state (*s*) at time (*t*). Based on the year in which a state adopts a partnership program, we categorize states into Partnership or LTCIP states (treatment group) and non‐LTCIP^22^ states (control group). The coefficient β_*1*_ (the DID estimator in this case) estimates the effect of the LTCIP on LTCI ownership and Medicaid uptake. The regression estimates control for additional state specific fixed effects (θ_*s*_) which eliminate time‐invariant differences among various states and year fixed effects (σ_*t*_) to flexibly account for variation across time. We consider some relevant controls X_*ist*_ which allow us to compare people living in different states as they differ in terms of socio‐economic characteristics.

### 3.4 Event Study Design

Together with the difference‐in‐differences specification, we use a panel event study design to estimate the impact of the inception of the LTCIP in a specific state.^23^ A panel event study design estimates the dynamic treatment effects before and after a relevant event (the introduction of a LTCIP program). It ensures that the identified effects are free from the negative weighting problem of TWFE. That is, we avoid the problems of averaging over heterogenous treatment effects when treatment occurs in different time periods for different units (L. Sun and Abraham 2020; Clarke and Schythe 2020). Additionally, it is designed to test if the parallel pre‐trend assumption of the Difference‐in‐Differences designs is satisfied by confirming whether the coefficients before the treatment are close to zero and statistically insignificant. More specifically, the event study specification we formulate is represented as follows:



(2)

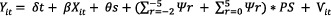




In Equation (2), δt and θs indicate year and state fixed effects, respectively. It must be noted that *r* = 0 corresponds to year 2006 that is, the interview was conducted 1 year after the adoption of DRA‐2005 after which the LTCIP programs were deployed across different states. Because HRS is a biennial survey, we do not observe the data recorded for year 2005. *X*_*it*_ indicates a vector of control variables and Ψr represents coefficients on leads and lags for Partnership states (PS) relative to the omitted category *Ψ*_−1_. Subsequently, we estimate Partnership group‐time average treatment effects, under the parallel trend and no anticipation effect assumptions, using an event study design for several group‐time combinations as suggested by Callaway and Sant’Anna (2021).

## 4 Results

### 4.1 Baseline Estimates

It is worth mentioning that the reported trends from Figure 1 do not control for time varying state‐level characteristics, alongside individual compositional differences. Hence, we estimate Equation (1), namely the Difference‐in‐Differences (DiD) design specification to identify the effect of LTCIP on LTCI ownership and Medicaid uptake. The baseline results reveal two main findings. First, the adoption of LTCIP increases the likelihood of LTCI purchase. Second, it subsequently reduces the likelihood of Medicaid take‐up.

Column (1) in Table 4 reports the baseline estimates of the impact of LTCIP programs on LTCI ownership drawn from a Two‐Way Fixed Effects model. We estimate a 1.5 percentage point (pp) increase in LTCI ownership,^24^ which entails an increase of appx. 14.7% in the LTCI ownership rate at the pre‐partnership average, or a LTCI‐ownership rate of 10.5% in the partnership states. Next, column (2) reports the estimates of the staggered rollout of the LTCIP program, and we re‐estimate the aggregate effect, using the method suggested by (Callaway and Sant’Anna 2021). Consistently, we find an estimated *effect of 1.75 pp increase in LTCI take up*. Both specifications incorporate a set of relevant control variables along with year and state fixed effects.

**TABLE 4:** Baseline results—Impact of partnership on LTCI and Medicaid.

	Dependent variables			
	LTCI (μ_pre‐policy_ = 0.105)		Medicaid (μ_pre‐policy_ = 0.062)	
	TWFE	Staggered DiD	TWFE	Staggered DiD
	(1)	(2)	(3)	(4)
Partnership (LTCIP)	0.0154***	0.0176***	−0.0082***	−0.0087***
	(0.0048)	(0.007)	(0.004)	(0.005)
State + Year fixed effects	YES	YES	YES	YES
Controls	YES	YES	YES	YES
*R*^2	0.035	0.035	0.141	0.141
*N*	156,102	156,102	156,102	156,102

*Note:* The estimates are retrieved using the sample from Health and Retirement Study, Waves 3–14, 1996–2018. We use two dependent variables namely Private long‐term care insurance (LTCI) and Medicaid. The variable ‘Partnership (or Post*Partnership‐states)’ is a treatment variable, which is a binary indicator for whether the state adopted Partnership program each year after the passage of Deficit Reduction Act (DRA‐2005). We refer to it either as ‘Partnership’ or ‘LTCIP’. At first, we estimate the impact of the LTCIP program on LTCI ownership in which Column (1) using the usual Two‐Way Fixed Effects model. Column (2) reports the estimates using a staggered Difference‐in‐Differences approach. Each coefficient indicates OLS estimates of the model specified in Equation (2). We control for demographic variables (namely age, gender, income, health status, marital status, race, and education), a set of chronic conditions (Cancer, diabetes, stroke, heart disease, lung disease, psychological disease, and arthritis) and ACA Medicaid expansion. Columns (3)–(4) repeats the similar procedure for Medicaid. Table S2 of the Appendix provides detailed baseline results.

*Significant at 10%; ** significant at 5%; *** significant at 1%, robust standard error clustered at state level. All the coefficient estimates are weighted using survey weights at person‐level.

Additionally, column (3) and (4) report analogous estimates using the same strategy as in Equations (1) and (2) but for Medicaid take‐up. Column (3) estimates a TWFE specification and finds that LTCIP reduces the likelihood of Medicaid uptake by 0.82 pp in the LTCIP states, which is appx. 13.3% decrease in Medicaid uptake rate compared to the pre‐partnership average Medicaid uptake rate of 8.6% in the LTCIP states. Similarly, column (4) reports the estimates retrieved using a staggered DiD model and consistently finds that the introduction of the LTCIP decreases the Medicaid take‐up by 0.87 pp. Although the coefficient for Medicaid uptake differs significantly from that of LTCI ownership, the results suggest that the introduction of the LTCIP program might have delayed the uptake of Medicaid in the states where the LTCIP program was implemented, thereby suggesting a 1‐to‐0.52 passthrough.

One possible explanation for the reduced Medicaid uptake after the implementation of the LTCIP program is that individuals who respond to the partnership program may be relatively more affluent and tend to over‐insure against average financial risks, thus not qualifying for Medicaid. However, the more likely explanation is that underwriting for LTCIP contracts may be less stringent, as Medicaid eventually covers the care costs if individuals need care for an extended period of time, hence Medicaid acts effectively as a catastrophic insurer. To explore this issue further we have examined the heterogeneous effects by population characteristics such as age, health status, retirement status, ethnicity, and presence of children all being criteria that could either influence the need of care, or be observables that insurance companies could use to exclude some individuals from receiving coverage.

Next, Table 5 reports treatment effect measures for various treatment comparisons as suggested by (Callaway and Sant’Anna 2021). More specifically, Table 5 reports the estimates of the impact of the LTCIP program on LTCI ownership and Medicaid uptake comparing various treatment groups to that of the never treated group. This comparison reveals that the introduction of the LTCIP program in a state increases LTCI ownership for all the treatment groups, but the estimates are only precisely estimated among the group of states that introduced the LTCIP after 2008 (Group‐2008) and 2010 (Group‐2010) respectively as most states are covered under these two time periods. Furthermore, we estimate that the introduction of the LTCIP program significantly reduced Medicaid uptake for two other groups of states, namely the Group‐2008 and Group‐2012. Hence, it seems that the effects for Medicaid are affected by the Great Recession, as the effect is then significant among the group of states that introduced the program after 2012 (Goroup‐2012).

**TABLE 5:** Group‐time effects—Impact of partnership on LTCI and Medicaid.

Panel A	LTCI ownership: Treated Vs never treated			
	(1)	(2)	(3)	(4)
	Gp2006	Gp2008	Gp2010	Gp2012
Partnership (LTCIP)	0.001	0.019***	0.018*	0.011
	(0.019)	(0.006)	(0.010)	(0.011)

Panel B	Medicaid uptake: Treated Vs never treated			
	(5)	(6)	(7)	(8)
	Gp2006	Gp2008	Gp2010	Gp2012
Partnership (LTCIP)	−0.005	−0.008*	−0.003	−0.028***
	(0.007)	(0.005)	(0.007)	(0.009)
State & year FE + controls	YES	YES	YES	YES
*N*	55,353	125,220	67,863	60,813
*N*_group_	4304	74,171	16,814	9764

*Note:* The estimates are retrieved using the sample from Health and Retirement Study, Waves 3–14, 1996–2018. Each coefficient refers to an OLS estimate of Equation (2). Panel A represents group‐time estimates of the impact of LTCIP program on LTCI ownership for Treated groups Versus Never Treated group. Each cohort of states adopting the LTCIP program in a specific year is compared with never treated states. Panel B reports the group‐time estimates of the impact of the state introduction of the LTCIP on Medicaid uptake for Treated groups Versus Never Treated group. All models are inclusive of state as well as year fixed effects and control variables (demographic controls, ACA Medicaid Expansion, & a set of chronic diseases).

*Significant at 10%; ** significant at 5%; *** significant at 1%, robust standard error clustered at state level. All the coefficient estimates are weighted using survey weights at person‐level.

### 4.2 Event Study

**FIGURE 2:**
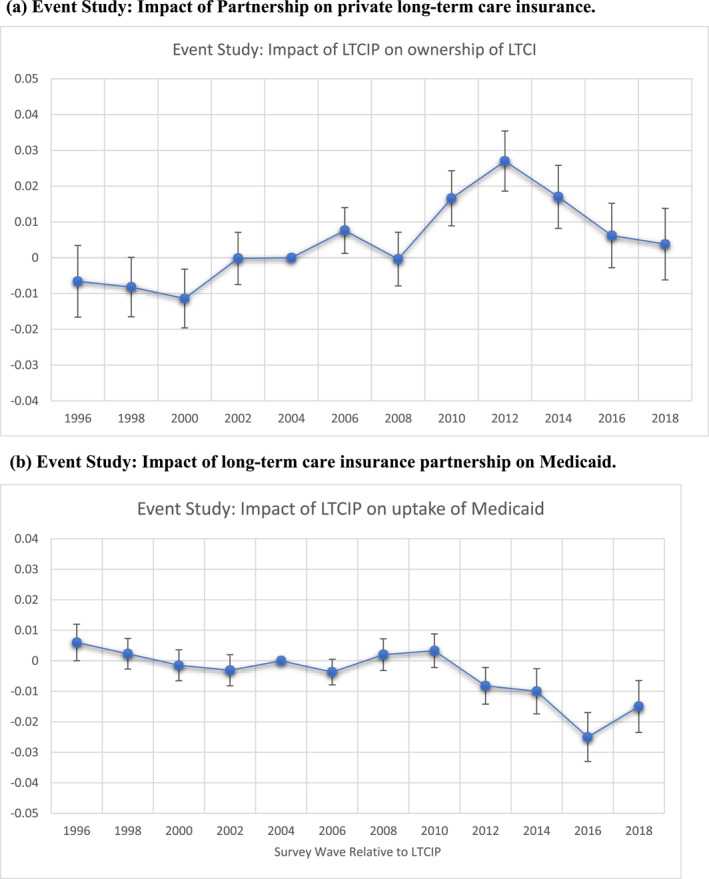
(a) Event Study: Impact of the Partnership (LTCIP) program on private long‐term care insurance. (b) Event Study: Impact of long‐term care insurance partnership on Medicaid. Each point in the Figure 2a,b indicates the effect of the partnership (LTCIP) program on LTCI and Medicaid relative to event time estimated using event study in Equation (1), with survey wave for the year 2006 reporting the partnership for the first time after DRA‐2005 is designated as year 2006. Given that the HRS is a biannual survey, the points on *X*‐axis are two years apart. The bars associated with each point on the plot represent standard errors associated with coefficient. All the coefficient estimates are weighted using survey weights at person‐level.

Figure 2a,b plots the estimated coefficients retrieved from event study regressions (Equation 1). The figures report the impact of the introduction of a LTCIP program on LTCI ownership and Medicaid uptake, and document that the effect of LTCIP on LTCI builds up over time and reaches a peak after 4–6 years (2–3 waves of the sample). Similarly, the effect of LTCIP on Medicaid take‐up follows subsequently from the impact of LTCIP on LTCI ownership. The parallel trend assumption is fully satisfied, and the estimates suggests that the LTCIP program increases LTCI ownership alongside the take‐up of Medicaid in the post‐DRA 2005 era. The same is true for LTCI ownership pre‐trends, as we conduct a pre‐trend test by testing the joint significant of coefficients in the pre‐partnership period. Table S4 in the Appendix reports the results of the test.^25^

### 4.3 Robustness Checks

The reported estimates are robust to various sets of robustness checks. First, we control for state tax subsidy on LTCI purchase and find similar and consistent estimates. These two programs were independently active at the same time, though a few states were exposed to both the LTCIP program as well as tax subsidy programs during the same period. The estimates including state specific tax subsidies are reported in Table 6 and suggest that the coefficient of LTCIP in a state increases LTCI ownership by 1.6 pp.^26^ Second, we ran our main specification including individual fixed effects which estimate within individual changes in the relevant outcomes. Our estimates suggest that incorporating individual level fixed effects does not modify our baseline estimates though it reduces the effect of LTCIP on LTCI ownership to 1.0 pp. However, in such a case the effect of Medicaid disapears as Medicaid eligibility is a one‐off event. Third, we test the robustness of our estimates to the use of non‐linear models. More specifically, we estimate a logit model specification and find that the reported average marginal effects are comparable to our baseline estimates from Equation (1), and although the effect of Medicaid decreases slightly it is consistent with our baseline estimates. Fourth, we add controls for wealth as well as household characteristics to Equation (1). Again, we find that these additions do not alter our baseline estimates. Fifth, we remove respondents with at least one chronic condition^27^ and re‐estimate Equation (1). As excepted, we observe that focusing only on individuals with no chronic condition increases the effect of LTCIP on LTCI ownership to 2.6 percentage points, whereas impact of Medicaid uptake remains almost unchanged.

**TABLE 6:** Robustness checks—The effect partnership on LTCI ownership & Medicaid updatake.

	Private LTCI	Medicaid
	(1)	(2)
μ_pre‐policy mean_	0.105	0.062
State + year FE	YES	YES
Controls	YES	YES
Controlling for tax‐subsidy		
Partnership (LTCIP)	0.016***	−0.008**
	(0.005)	(0.004)
Tax subsidy	0.005	−0.001
	(0.004)	(0.003)
Individual fixed effects model		
Partnership (LTCIP)	0.01***	−0.0001
	(0.003)	(0.002)
Logit model (AMEs)		
Partnership (LTCIP)	0.0152***	−0.006*
	(0.047)	(0.003)
Addition of wealth as a control variable		
Partnership (LTCIP)	0.0165***	−0.009**
	(0.048)	(0.004)
*N*	156,102	156,102
Removing respondents with chronic diseases		
Partnership (LTCIP)	0.026***	−0.008**
	(0.007)	(0.004)
*N*	78,912	78,912
Addition of household level control (number of children)		
Partnership (LTCIP)	0.015***	−0.008**
	(0.048)	(0.004)
*N*	153,710	153,710
Control group: Never‐treated states only		
Partnership (LTCIP)	0.011**	−0.007*
	(0.006)	(0.004)
*N*	125,771	125,771
Control group: Permanent partnership states only		
Partnership (LTCIP)	0.017***	−0.008**
	(0.0053)	(0.004)
*N*	135,384	135,384

*Note:* The estimates are obtained using the sample from Health and Retirement Study, Waves 3–14, 1996–2018. Each coefficient indicates OLS estimates of Equation (2). Each category title on the left‐hand side of the table refers to a specification change incorporated to check if the baseline model estimates are robust to change in specifications. All models are inclusive of state as well as year fixed effects and control variables (demographic controls, ACA‐Medicaid, and a set of chronic diseases).

*Significant at 10%; ** significant at 5%; *** significant at 1%, robust standard error clustered at state and household level. All the coefficient estimates are weighted using survey weights at person‐level.

Lastly, we examine the sensitivity of our estimates to distinguishing the control group into two groups, namely ‘Partnership states Versus Never‐Treated states’ (Sample I) and ‘Partnership states Versus Permanent Partnership states’ (Sample II). When we run our baseline specification on these two samples separately, we find that compared to respondents living in Never‐Treated states, the LTCIP state respondents are 1.13 percentage points more likely to own a LTCI policy and 0.67 percentage points less likely to take‐up Medicaid. Similarly, when we restrict the control group to permanent partnership states alone, we find an effect that compares to our baseline estimates. That is, we estimate that the impact on LTCI ownership increases to 1.69 percentage points and a reduction in Medicaid take‐up of 0.84 percentage points. So, these results suggest that the introduction of LTCIP program in a state increases the likelihood of LTCI purchase compared to respondents in permanent partnership states.

### 4.4 Heterogeneity

Table 7 shows how heterogeneous the estimates are to different individual characteristics and the level of pre‐partnership LTCI ownership. The adoption of the LTCIP programs differs across states, with some states adopting the LTCIP program immediately after the passage of Deficit Reduction Act (2005), whereas other followed a few years later.^28^ The use of HRS data provides an advantage to examine how our estimates vary across different sub‐populations. To such end, we estimate the fully specified models to find how LTCI ownership and Medicaid uptake respond to the adoption of a LTCIP in a state across different observable characteristics such as age, health status, gender, education, wealth level, retirement status, marital status, children, and ethnicity. We observe that the effect of LTCIP program on LTCI ownership is concentrated among individuals who are married, educated, retired, white, and those with income level above median. In contrast, the effect on Medicaid uptake is stronger among the individuals in poorer health, non‐whites, those un‐married or single, and those without children.

**TABLE 7:** Heterogeneity in the effect of LTCIP on LTCI and Medicaid (interaction effects).

		Private LTCI	Medicaid
		(1)	(2)
μ_pre‐policy mean_		0.105	0.062
State & year FE		YES	YES
Controls		YES	YES
*N*_Total_		156,102	156,102
Income	Above median (*N* = 78,051)	0.017*** (0.006)	−0.008** (0.003)
	Below median (*N* = 78,051)	0.008* (0.005)	−0.002 (0.006)
Health	Good/Best/Excellent (*N* = 114,885)	0.015*** (0.005)	−0.004 (0.004)
	Fair/Poor (*N* = 41,217)	0.016*** (0.006)	−0.023*** ϮϮϮ (0.01)
Gender	Female (*N* = 88,662)	0.013** (0.006)	−0.008* (0.004)
	Male (*N* = 67,440)	0.018*** (0.006)	−0.009** (0.004)
Education	High school/Less (*N* = 84,087)	0.008 (0.005)	−0.009* (0.005)
	Some/More college (*N* = 72,015)	0.02***Ϯ (0.006)	−0.008** (0.004)
Wealth	Low (< $149K; *N* = 78,113)	0.007 (0.005)	−0.01* (0.005)
	Middle ($149K–$540K; *N* = 46,771)	0.015** (0.007)	−0.008** (0.004)
	High (> $540K; *N* = 31,218)	0.029*** ϮϮ (0.010)	−0.001 ϮϮ (0.003)
Retirement status	Working (*N* = 63,118)	0.009 (0.007)	−0.007** (0.004)
	Retired (*N* = 73,506)	0.025*** ϮϮ (0.006)	−0.010** (0.005)
Marital status	Not‐married (*N* = 53,341)	0.008 (0.006)	−0.012** (0.006)
	Married (*N* = 102,761)	0.019*** (0.006)	−0.006* (0.004)
Have children	NO (*N* = 10,853)	0.019 (0.016)	−0.027*** (0.009)
	YES (*N* = 142,773)	0.015*** (0.005)	−0.006* ϮϮ (0.004)
ACA‐medicaid expansion	NO (*N* = 135,570)	0.020*** (0.005)	−0.004 (0.003)
	YES (*N* = 20,532)	0.007 (0.008)	−0.017*** (0.006)
Ethnicity	Non‐white (*N* = 39,927)	0.0108 (0.007)	−0.017** (0.008)
	White (*N* = 116,175)	0.016*** (0.005)	−0.007* (0.004)

*Note:* The estimates are obtained using the sample from Health and Retirement Study, Waves 3–14, 1996–2018. Each coefficient indicates OLS estimates for outcomes private long‐term care insurance and Medicaid. Robust standard errors are clustered at state level. Each category on the left‐hand side of the table indicates a separate regression that includes interactions between subgroup indicators and treatment variable (Partnership or LTCIP). All models are inclusive of state as well as year fixed effects and control variables (demographic controls and a set of chronic diseases). All the coefficient estimates are weighted using survey weights at person‐level.

*denotes significantly different from zero (* significant at 10%; ** significant at 5%; *** significant at 1%); Ϯ denotes that bottom category estimates are significantly different from top category ones (Ϯ significant at 10%; ϮϮ significant at 5%; ϮϮϮ significant at 1%).

The effect of LTCIP program adoption by a state on LTCI ownership is larger among highly educated individuals compared to less‐educated ones, yet a similar effect is observed when we examine the effect on Medicaid take‐up. Consistently, the LTCIP increases the LTCI ownership as an individual climbs‐up the wealth ladder. In contrast, we find a statistically significant decrease in Medicaid uptake among relatively wealthier individuals before they need care. We have also checked if ACA‐Medicaid had any impact on our estimates. We do so by interacting our treatment variable with a dummy variable identifying states that participated in the ACA‐Medicaid expansion after 2014. We find that states that introduced a LTCIP program and also expanded Medicaid after 2014 exhibit an impact on LTCI ownership that was not statistically significant from zero, and the effect size was also negligible compared to LTCIP states which did‐not expand Medicaid until 2018 suggesting that the effect of LTCIP on LTCI purchase is not driven by ACA Medicaid expansion after 2014. In contrast, we do find a stronger effect of LTCIP on Medicaid uptake among those states expanding Medicaid after 2014. Such an effect on Medicaid uptake is consistent with the evidence from Figure 2b suggesting that the effect of LTCIP on Medicaid uptake after 2014 is declined more intensively than earlier years.

The LTCIP effect on LTCI ownership is driven by retired individuals, as we find no statistically significant effect for working individuals. Similarly, the impact on Medicaid uptake was greater in magnitude among retired individuals. We find that the LTCIP effect is stronger among married individuals, but the impact on Medicaid uptake is more pronounced among non‐married individuals who lack informal support within the family (Costa‐Font 2010; Costa‐Font and Courbage 2015). We also document that, as expected, individuals without children are more likely to purchase LTCI, compared to those with children, and the effect on Medicaid is also stronger for these individuals. Finally, we find that LTCI ownership increased among white Americans compared to other minorities, whereas the opposite effect is observed for Medicaid uptake.

## 5 Medicaid Savings Simulation After the Adoption of Partnership

Next, we estimate the expected savings to Medicaid resulting from the introduction of the LTCIP, which we anticipate to increase LTCI coverage, particularly among middle‐wealth households (Lin and Prince 2013; Bergquist, Costa‐Font, and Swartz 2018). The effects on Medicaid are expected as LTCIP conveyed extra benefits via additional wealth protection due to higher asset thresholds for Medicaid eligibility, which prevents spending‐down effects (Pauly 1990). Consistently, previous evidence indicates that absence of LTCI ownership can lead to increased self‐insurance in the form of out‐of‐pocket expenses and, when individuals are eligible for Medicaid, can result in higher public expenditure (Brown and Finkelstein 2007, 2008, 2011; Goda 2011; Bergquist, Costa‐Font, and Swartz 2018; Frank 2012). Hence, it is important to evaluate whether the introduction of the LTCIP exerted an effect on Medicaid expenditure. To this end we implanted a simple simulation model, in line with that of Goda (2011), to predict the impact of partnership on fiscal public Medicaid expenditure.^29^

### 5.1 Simulation Procedures

We simulate the impact of adopting partnership programs for a 65‐year‐old with gender *g* and wealth decile *I*, following Goda (2011)'s simulation model for tax subsidy. We define *C*_*i*_(I) and *C*'_i_(*I*) = *C*_*i*_(*I*) + *P*_*i*_ as a coverage rate of LTCI before and after the adoption of partnership, respectively, in which P_*i*_ is the change in LTCI coverage due to partnership. The Medicaid share of the expected present discounted value (EPDV hereafter) of long‐term care expenditures for gender *g* and wealth decile *i,* with and without LTCI coverage, are denoted by *M*_*i,g*_(*I*)^30^ and *M*_*i,g*_(*N*) (*M*'_*i,g*_(*I*) for Partnership), respectively. Let *M*_*i,g*_(*P*) and *M*'_*i,g*_(*P*) be the share of Medicaid before and after the adoption of partnership program. We obtain the Medicaid share of EPDV for LTC for a non‐partnership plan from (J. R. Brown and Finkelstein 2008). For partnership plan, the EPDV share of Medicaid is calculated, using the treatment effects across wealth levels, and presented in Table 8. They are defined as:

(3) $$M_{i,g}\left(P\right)=C_{i}\left(I\right)\;*\;M_{i,g}\left(I)+(1-C_{i}\left(I\right)\right)*\;M_{i,g}\left(N\right)$$

(4) $$M_{i,g}^{\prime }\left(P\right)=C_{i}^{\prime }\left(I\right)\;*\;M_{i,g}^{\prime }\left(I)+(1-C_{i}^{\prime }\left(I\right)\right)*\;M_{i,g}\left(N\right)$$

**TABLE 8:** Share of EPDV of long‐term care expenditure paid for by Medicaid.

Wealth % ile	Medicaid share of EPDV					
	Men			Women		
	No insurance	insurance	Partnership Insurance	No insurance	Insurance	Partnership insurance
10th	0.98	0.52	0.56	0.99	0.55	0.59
20th	0.89	0.44	0.47	0.93	0.50	0.54
30th	0.80	0.41	0.44	0.88	0.46	0.50
40th	0.71	0.37	0.40	0.80	0.43	0.46
50th	0.60	0.32	0.34	0.72	0.38	0.41
60th	0.46	0.26	0.30	0.60	0.33	0.38
70th	0.32	0.20	0.23	0.45	0.24	0.28
80th	0.17	0.12	0.16	0.24	0.15	0.20
90th	0.07	0.05	0.07	0.08	0.06	0.08
Average	0.56	0.30	0.33	0.63	0.34	0.38

*Note:* The estimates for Medicaid share of EPDV with and without LTCI are obtained from J. R. Brown and Finkelstein (2008). The EPDV share of Medicaid for partnership insurance is calculated after multiplying the effect of LTCIP program on LTCI across various wealth quintiles (Quintiles: 1–5, Low Wealth (β_LTCI_ = 0.0072); 6–8, Middle Wealth (β_LTCI_ = 0.015**); 9–10, high Wealth individuals (β_LTCI_ = 0.029***)). For example, for the 40th percentile of wealth, the EPDV share of Medicaid for partnership = (1 + 0.0072*0.107*100) *(EPDV share of Medicaid for regular insurance policy (J. R. Brown and Finkelstein (2008))). Where, 0.107 is the average take up of LTCI in the sample, 0.0072 is β_LTCI_ for low wealth individuals. The EPDV of total long‐term care expenditures used for simulation analysis calculations, for women and men, are $52,523 and $21,021 respectively. The estimates of Partnership for Medicaid share of EPDV are the most conservative estimates used for the calculation of Medicaid savings in our simulation analysis.

Let *E*_*g*_(LTC) be the EPDV of long‐term care costs for a person with gender *g.* Therefore, the expected Medicaid savings, $E_{i,g}\left(S\right)$, due to the adoption of LTCIP program for an individual of gender *g* and wealth decile *i* are as follows:

(5) $$E_{i,g}\left(S\right)=\left(\;M_{i,g}\left(P\right)-M_{i,g}^{\prime }\left(P\right)\right)*\;E_{g}\left(\text{LTC}\right)-E\left(C\right)$$

where *E*(*C*) refers to the expected cost of implementation of partnership program per person. The program implementation cost does not differ for individuals gender *g* and wealth decile *i. In other words, the cost is the same for all individuals.* However, we assume that the implementation of the LTCIP program incurs little to no costs. Thus, while calculating and reporting the expected Medicaid savings, we insert *E*(*C*) = 0 in Equation (5).

### 5.2 Simulation Assumptions

We use the above model to predict Medicaid expenditure savings after the adoption of the partnership program. However, we make some assumptions concerning the effect of the adoption of the partnership program (LTCIP) on LTCI ownership rates, premiums, and Medicaid costs (Goda 2011). Column (2) of Table S5 (Appendix) indicates our assumptions with respect to the impact of the LTCIP program by Low, Middle, and High wealth levels correspond to 30th, 60th, and 80th percentile of wealth, respectively. We also linearly interpolate responses for the remaining percentiles. Following Goda (2011), we use the estimates of *M*_*i,g*_(*I*) and *M*_*i,g*_(*N*) which depict the Medicaid share of EPDV for LTCSS by gender *g* and wealth decile *i* for 65‐year‐old individuals with and without private LTCI coverage, provided by Brown and Finkelstein (2008). We use an annual premium of *θ* = $2000—which is gender neutral—and assume that LTCI coverage provides a daily benefit of $100 for a 65‐year‐old individual. Brown and Finkelstein (2008) and Goda (2011) use similar estimates of the EPDV of LTC costs by gender, for the year 2000. The estimate *E*_*f*_(LTC) = $43,750 for women and *E*_*m*_(LTC) = $17,500 for men. However, we calculate these values of EPDV for the year 2006. Thus, our estimates are updated to *E*_*f*_(LTC) = $52,523 for women and *E*_*m*_(LTC) = $21,021 for men, in our simulation model.

### 5.3 Simulation Results

**FIGURE 3:**
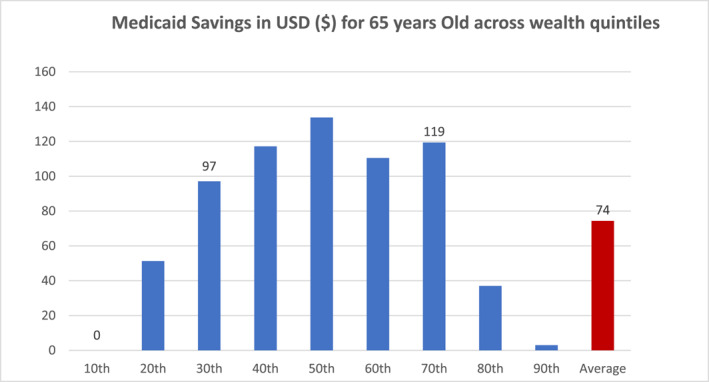
The figure depicts the estimated total net savings from LTCIP, for a 65 year old individual by wealth deciles, adjusted for the increase in Medicaid share of EPDV post‐reform. These saving estimates are calculated using the estimated effects across wealth levels obtained from Column (2) of Table 7 and using the LTCIP program increase in Medicaid share of EPDV from the Table 9. Average Medicaid savings for a 65 years old are calculated using a simulation technique that follows Goda (2011). Authors calculate, with reference to year 2006, the expected present discounted value (EPDV) of long term‐care costs or E(LTC) of $21021 for men and $52523 for women, using the values assumed by Brown and Finkelstein (2007) and Goda (2011) for the year 2000. Low, Middle, and High wealth levels correspond to 30th, 60th, and 80th percentile respectively. The horizontal axis represents wealth percentiles, and the vertical axis represents amount saved in US$.

If the implementation of LTCIP incurs little to no administrative costs, Figure 3 reports the estimated net Medicaid savings across different levels of wealth. As expected, we estimate savings for an individual at the 10th percentile of wealth to be zero but savings increase for the 20th, 30th, and 40th percentile to be $51, $97, and $117 respectively. Again, given that by design, low income poeple qualify for Medicaid, the 90th percentile corresponds to the lowest net saving after the 10th percentile. Consistently with the idea that LTCIP benefit middle income individuals, the net savings peak at the 50th percentile at a value of $134 and they decline as the wealth percentile increases. Its worth noting that the 50th to 70th percentile corresponds to $149,000 to $343,000 of total assets, which fall within the GAO's assumed assets range ($100,000–$350,000) for Middle‐Middle group individuals. Unsurprisingly, the net Medicaid savings is only a negligible magnitude of $3 at the high end of the wealth distribution (the 90th percentile). Overall, the estimates suggest Medicaid savings to be on average $74 per 65‐year‐old. However, the 95% confidence interval ranges from $20 to $128. Overall, we conclude that an increase in LTCI ownership after the adoption of the LTCIP program by a state can potentially influence government Medicaid savings.

Our estimates can be explained as follows. First, individuals with below the media level of wealth are likely to accumulate fewer savings after participating in the LTCIP, as more people in these groups tend to rely on Medicaid once their savings are exhausted, compared to those above the median level of wealth. Second, savings begin to decline as individuals move up the wealth distribution, particularly after the 50th percentile. This can be attributed to the stigma surrounding Medicaid among wealthier individuals, especially in conservative states, where negative views about public insurance programs discourage potential beneficiaries from Medicaid coverage (Sommers et al. 2012; Allen et al. 2014). In addition, we find that as expected the higher uptake of LTCI ownership for high‐wealth individuals does not significantly alter their Medicaid expenditure for long‐term care (Goda 2011). Our findings also indicate that although the response to LTCIP is strongest among high‐wealth individuals, these groups contribute to lower Medicaid savings too, as there is barely no significant change in their propensity to take up Medicaid possibly due to Medicaid stigma.

### 5.4 Sensitivity Analysis

Consistent with Goda (2011), we perform a further sensitivity analysis on to estimate Medicaid savings for 65‐year‐old individuals. As part of the sensitivity analysis, we first calculate the expected long‐term care costs (*E*_*g*_(LTC) or EPDV) at a 10% tolerance in both directions and then replace the EPDVs from our main simulation with the adjusted ones for 65‐year‐old individuals with gender *g*. The results from Table 9 indicate that changing EPDVs at a 10% tolerance level alters the Medicaid savings by $7 above and below the baseline value of $74. Medicaid savings at +10% and −10% of EPDV are $81 and $67, respectively.

**TABLE 9:** Sensitivity analysis for simulation output.

		Total Medicaid savings
Main model	Baseline	$74
	+2SD	$128
	−2SD	$20
Eg(LTC)	1.1*EPDV (or +10%)	$81
	0.9*EPDV (or −10%)	$67

*Note:* The expected present discounted value (EPDV) of long term‐care costs or E(LTC) for men (women) is $21021 ($52523).

### 5.5 LTCIP Benefits and Target Efficiency

One of the major reasons for low LTCI ownership in the US is the secondary payer status of Medicaid, which imposes an implicit tax on LTCI (Brown and Finkelstein 2008, 2011). Medicaid's implicit tax on LTCI can be partially eliminated, by delaying the process of qualifying for Medicaid, via the adoption of LTCIP program (Brown and Finkelstein 2011). Thus, the effect of LTCIP program  adoption must also be looked through the lens of ordeals. The main purpose of ordeals is to achieve target efficiency by reaching out to those who need it the most (Nichols and Zeckhauser 1982; Zeckhauser 2021). Consistently, Table 10 outlines four types of potential Medicaid beneficiaries resulting from the introduction of the LTCIP program, labeled A, B, C, and D. As individuals become more affluent, they tend to purchase higher amounts of coverage. If everyone fully protects their remaining assets after paying the LTCIP premium, the primary goal of the partnership program is to serve individuals in group D. Indeed, individuals in group B being relatively wealthier, are more likely to have greater coverage, with most of their long‐term services and supports (LTSS) expenses financed by private insurance. Similarly, individuals in group A and C require only partial LTSS support, which is covered by the LTCIP program, sparing them from having to qualify for Medicaid. Thus, the LTCIP program can achieve target efficiency even in the presence of Medicaid's implicit tax on LTCI.

**TABLE 10:** Intended and actual beneficiary of Medicaid via partnership.

Class	Some LTSS	Full LTSS
Affluent	A	B
Middle Income	C	D

## 6 Conclusion

This paper has examined whether the rollout of the Long‐Term Care Insurance Partnership (LTCIP) program in the United States has managed to successfully increase long term care insurance (LTCI) ownership and slowed down Medicaid uptake, as intended by the program. Our estimates draw on more than two decades worth of data from the Health and Retirement Study (from 1996 through 2018) and take advantage of recent developments in a generalized DiD design to exploit the progressive adoption of partnership over time after the passage of the federal Deficit Reduction Act (DRA) in 2005.

We document robust evidence of a moderate increase in LTCI ownership after the introduction of the LTCIP program, in contrast to previous studies that focused on the short‐term effects of the early states implementing the program. Our preferred estimates indicate that the LTCIP rollout led to a 1.54 pp increase in LTCI ownership and a 0.82 pp reduction in Medicaid uptake. Furthemore, evidence from our simulation analysis suggests that the introduction of the LTCIP program gave rise to an average $74 in Medicaid savings per 65‐year‐old. Although the effect on LTCI ownership is smaller in magnitude, it appears to significantly reduce the uptake of Medicaid, leading to non‐negligible savings in Medicaid spending. Such significant savings are explained by the relatively low implementation costs of the LTCIP for state governments, and the fact that the LTCIP enables middle‐income individuals to purchase insurance coverage. Such individuals would otherwise be covered by Medicaid after spending down their wealth. Our results suggest that the introduction of a LTCIP program in a state did seem to delay Medicaid uptake by incentivizing the purchase of LTCI. Furthermore, our estimates suggest *that the implicit tax on LTCI resulting from the Medicaid program design can be reduced by the LTCIP program*. Most importantly, our findings certainly create avenues for other potential insurance designs that expand and complement the effects of the LTCIP program.

## Conflicts of Interest

The authors declare no conflicts of interest.

## Supporting information

Supporting Information S1

## Data Availability

The data that support the findings of this study are available on request from the corresponding author. The data are not publicly available due to privacy or ethical restrictions.

## Endnotes

^1^
The policy holders of LTCI can receive long‐term care services in‐house, in a nursing care centre, in an adult day‐care centre, or in an assisted living facility, and get the reimbursement for the money spent on buying such services.

^2^
Goda (2011) examines how tax subsidies impact LTCI ownership showing that increased LTCI ownership could reduce Medicaid spending on LTCSS. More specifically, 1 percentage point increase in LTCI ownership could result in potential Medicaid savings of $24 to $29 million annually. Furthermore, the secondary payer status of Medicaid imposes an implicit tax on LTCI leading to a reduction in the net benefits obtained from a private policy (Brown and Finkelstein 2008, 2011).

^3^
Although the typical stand‐alone LTCI policy introduced in 2010 remained stable for more than 5 years with no change in daily benefit amount and policy deductible period, its average annual premium has increased by almost 20% at the same time (Ameriks et al. 2016).

^4^
Adverse selection happens when those who anticipate needing long‐term care are more likely to buy insurance, which raises the costs of the insurance for the insurers and raises premiums as a result.

^5^
Figures S1 and S2 of the Appendix shows the average rate of LTCI ownership over time. Figure 1a depicts average LTCI ownership over time for partnership states, never‐treated states, and permanent partnership states.

^6^
The partnership program is administered through the combined effort of public and private insurance providers in the form of a new insurance product known as partnership (Robert Wood Johnson Foundation (RWJF) 2007; Lin and Prince 2013).

^7^
The Deficit Reduction Act (DRA) of 2005 was a bill that aimed to reduce the federal government's budget deficit and included measures to slow spending on Medicare and Medicaid.

^8^
Moreover, it is important to note that LTCIP supplemented existing state‐level fiscal incentives, many of which were not estimated to be cost‐effective (Goda 2011). Nevertheless, the return on investment of the partnership remains unknown.

^9^
The consideration of long term effects as individuals need some time to learn about the program and to switch to a LTCIP policy from a standard one.

^10^
As of January 2019, the income eligibility criteria to qualify for Medicaid is 138% of the federal poverty line.

^11^
The expansion of LTCIP was the result of the DRA recommendations in 2005, allowing individuals to replace traditional insurance policies with partnership policies after the introduction of the LTCIP program in a given state.

^12^
Given the protective wealth effects of the partnership program, we expect such DRA2005 clause to increase the likelihood that a policyholder will hold LTCI insurance.

^13^
Although the ‘dollar‐for‐dollar’ model was initiated by California, Connecticut, and Indiana and later embraced by New York in 2006 (M. R. Meiners, McKay, and Mahoney 2002; NYSPLTC 2011; Bergquist, Costa‐Font, and Swartz 2018), all partnerships developed after 2006 follow the ‘dollar‐for‐dollar’ model by default.

^14^
In the absence of LTCIP, however, individuals must spend down their assets to meet Medicaid's strict eligibility thresholds, and any remaining assets may be subject to estate recovery in some states, potentially depleting their estate and reducing what is passed on to the next generation or a spouse.

^15^
We use HRS data, as compared to Panel Study of Income Dynamics (PSID) data, contains detailed longitudinal information on both supply and demand for long‐term care services, including long‐term care insurance, and focuses on individuals in the age groups that are more likely to both purchase and use LTCI. Unlike PSID, HRS captures critical aspects of aging, including financial preparedness for long‐term care needs, which is a key for understanding how a typical household navigate these challenges.

^16^
The analytical approaches used in Brown, Coe and Finkelstein (2007), Courtemanche and He (2009) and Goda (2011) focus on individuals aged 50–69. Therefore, our analytical sample does not deviate much from the existing studies and at the same time covers majority of the populaltion with a LTCI policy.

^17^
Based on the estimates from the existing studies, the attrition rate for HRS falls between 6% and 15% (Kapteyn et al. 2006; Banks, Muriel, and Smith 2011; Michaud et al. 2011), the response rate for core interviews ranges from 47.1% to 81.3%, and the re‐interview response rates range from 68.8% to 92.3% (Fisher and Ryan 2018). Attrition, if not random, can generate biased estimates in regression models if the attrition is related with LTCI ownership or Medicaid uptake studied here. Additionally, such attrition can threaten the external validity of the study if not addressed with properly. 

^18^
This is consistent with Ameriks et al. (2016) who reports that there was a rapid decline in the number of insurance providers in the market between 2002 and 2014 with many providers exiting market due to lack of sale of such policies.

^19^
One particular concern is that LTCIP are specific to certain companies. Cohen, Kaur, and Darnell. (2013), Brewster and Gutterman (2014) and Ameriks et al. (2016) discuss the exit of insurance providers and do not suggest evidence of this phenomena, and checks on the websites of insurance companies offering LTCI companies suggest that they had operations in both partnership as well as non‐partnership states.

^20^
That said, we observe a steep decline in LTCI ownership between 2014 and 2016 which might suggest a role of ACA‐Medicaid expansion, but we do not find any statistically significant evidence of this in our analysis we carry out later in the paper.

^21^
We did consider using the stacked DID model incorporated by Deshpande and Li (2019), which exploit the timing of the closings by considering only those zip codes that experienced closings and by dividing them into Current closings (treatment) versus future closings (control ‐ closings happen after 2 years), but this approach is not suitable for partnership due to limitation of biennial nature of HRS survey and the timing of partnership adopted by states. For example, most states adopted partnership between 2006 and 2010, which leaves us comparing 2008 states (treatment) versus 2010 states (control) for only one wave. This comparison is also not covered, in the subsequent section when we incorporate the method suggested by Callaway and Sant’Anna (2021), due to the same reason.

^22^
The control group consists of non‐partnership states that include never‐treated states (permanent‐partnership and other remaining states).

^23^
A growing number of studies have tested a combination of complex identifying assumptions in this regard and attempts to provide a guidance on accurately estimating the impact of staggered adoption of policies (Athey and Imbens 2021; Borusyak and Jaravel 2017; Callaway and Sant’Anna 2021; de Chaisemartin and D'Haultfoeuille 2020; L. Sun and Abraham 2020; Goodman‐Bacon 2021).

^24^
A theoretical model presented by W. Sun and Webb (2024) finds that LTCIP predicted to induce non‐purchasers aged 65 and above to purchase the coverage of LTCI by 4% among women and 2% among men, respectively. We find that LTCIP increased on LTCI coverage by 1.54 percentage points, which is equivalent to 14.7% the pre‐partnership average LTCI‐ownership rate of 10.5% in the partnership states, for individuals aged 50–75, as that's the premium age of purchasing LTCI. Thus, our main effect, is comparable to W. Sun and Webb (2024), though arguably their estimate is a lower bound for the age range we consider.

^25^
We find that all the coefficients are close to zero and statistically insignificant. Additionally, the *p*‐value of joint test is more that 50%, indicating that we fail to reject null hypothesis that coefficients are jointly zero.

^26^
The list of states providing tax‐subsidy are reported in Table S7 of the Appendix.

^27^
List of Chronic diseases considered: Diabetes, stroke, cancer, heart disease, lung disease, cancer, and a psychological disease.

^28^
Previous studies such as Bergquist, Costa‐Font, and Swartz. (2018) used the data on insurance contracts provided by National Association of Insurance Commission (NAIC), which do not include individual‐level information.

^29^
It is important to note that our estimates do not explicitly allow for Medicaid savings due to reduced asset transfer.

^30^
We test the change in EPDV of Medicaid share with insurance (*M*_*i,g*_(*I*)) for partnership in the sensitivity analysis and the results are reported in Table 9.

## References

[hec4949-bib-0001] AALTCI . 2019. American Association for Long‐Term Care Insurance. http://www.aaltci.org/.

[hec4949-bib-0002] AARP . 2019. “Does Medicare Cover Long‐Term Care, Nursing Home Care or Care in Skilled Nursing Facilities?” American Association of Retired Persons (AARP). https://www.aarp.org/health/medicare‐qa‐tool/current‐long‐term‐nursing‐home‐coverage/.

[hec4949-bib-0003] Ai, C. , and E. C. Norton . 2003. “Interaction Terms in Logit and Probit Models.” Economics Letters 80, no. 1: 123–129. 10.1016/S0165-1765(03)00032-6.

[hec4949-bib-0004] Allen, H. , B. J. Wright , K. Harding , and L. Broffman . 2014. “The Role of Stigma in Access to Health Care for the Poor: The Role of Stigma in Access to Health Care for the Poor.” Milbank Quarterly 92, no. 2: 289–318. 10.1111/1468-0009.12059. 24890249 PMC4089373

[hec4949-bib-0005] Alper, J. 2006. “The Partnership for Long‐Term Care: A Public‐Private Partnership for Financing Long‐Term Care.” Robert Wood Johnson X (To Improve Health and Healthcare).

[hec4949-bib-0006] America's Health Insurance Plans . 2007. Who Buys Long‐Term Care Insurance? A 15‐year Study of Buyers and Non‐buyers, 1990–2005. Prepared for AHIP by Lifeplans, Inc.

[hec4949-bib-0007] Ameriks, J. , D. Karapiperis , V. Bodnar , et al. 2016. “The State of Long‐Term Care Insurance: The Market, Challenges and Future Innovations.” National Association of Insurance Commissioners and the Center for Insurance Policy and Research. https://content.naic.org/sites/default/files/inline‐files/cipr_current_study_160519_ltc_insurance.pdf.

[hec4949-bib-0009] Athey, S. , and G. W. Imbens . 2006. “Identification and Inference in Nonlinear Difference‐In‐Differences Models.” Econometrica 74, no. 2: 431–497. 10.1111/j.1468-0262.2006.00668.x.

[hec4949-bib-0010] Athey, S. , and G. W. Imbens . 2021. “Design‐Based Analysis in Difference‐In‐Differences Settings With Staggered Adoption.” Journal of Econometrics 226, no. 1: 62–79. 10.1016/j.jeconom.2020.10.012.

[hec4949-bib-0011] Banks, J. , A. Muriel , and J. P. Smith . 2011. “Attrition and Health in Ageing Studies: Evidence From ELSA and HRS.” Longitudinal and Life Course Studies 2, no. 2. 10.14301/llcs.v2i2.115 PMC3872999 24376472

[hec4949-bib-0012] Bergquist, S. , J. Costa‐Font , and K. Swartz . 2018. “Long‐Term Care Partnerships: Are They Fit for Purpose?” Journal of the Economics of Ageing 12, no. November: 151–158. 10.1016/j.jeoa.2018.03.006.

[hec4949-bib-0013] Borysyak, K. , and X. Jaravel . 2017. “Revisiting Event Study Designs, With an Application to the Estimation of the Marginal Propensity to Consume.” SSRN Working Paper.

[hec4949-bib-0014] Brewster, R. , and S. Gutterman . 2014. The Volatility in Long‐Term Care Insurance. Society of Actuaries.

[hec4949-bib-0015] Brown, J. R. , and A. Finkelstein . 2007. “Why Is the Market for Long‐Term Care Insurance So Small?” Journal of Public Economics 91, no. 10: 1967–1991. 10.1016/j.jpubeco.2007.02.010.

[hec4949-bib-0018] Brown, J. R. , and A. Finkelstein . 2008. “The Interaction of Public and Private Insurance: Medicaid and the Long‐Term Care Insurance Market.” American Economic Review 98, no. 3: 1083–1102. 10.1257/aer.98.3.1083.

[hec4949-bib-0019] Brown, J. R. , and A. Finkelstein . 2011. “Insuring Long‐Term Care in the United States.” Journal of Economic Perspectives 25, no. 4: 119–142. 10.1257/jep.25.4.119.

[hec4949-bib-0020] Brown, J. R. , G. S. Goda , and K. McGarry . 2012. “Long‐Term Care Insurance Demand Limited by Beliefs About Needs, Concerns About Insurers, and Care Available From Family.” Health Affairs 31, no. 6: 1294–1302. 10.1377/hlthaff.2011.1307. 22665842

[hec4949-bib-0017] Brown, J. R. , N. B. Coe , and A. Finkelstein . 2007. “Medicaid Crowd‐Out of Private Long‐Term Care Insurance Demand: Evidence From the Health and Retirement Survey.” Tax Policy and the Economy 21: 1–34. 10.1086/tpe.21.20061913.

[hec4949-bib-0021] Callaway, B. , and P. H. C. Sant’Anna . 2021. “Difference‐in‐Differences With Multiple Time Periods.” Journal of Econometrics 225, no. 2: 200–230. 10.1016/j.jeconom.2020.12.001.

[hec4949-bib-0022] Clarke, D. , and K. Schythe . 2020. Implementing the Panel Event Study: IZA DP No. 13524.

[hec4949-bib-0023] CMS . 2018. Centers for Medicare & Medicaid Services. https://www.cms.gov/. 26110197

[hec4949-bib-0024] Cohen, M. A. , R. Kaur , and B. Darnell . 2013. “Exiting the Market: Understanding the Factors Behind Carriers’ Decision to Leave the Long‐Term Care Insurance Market.” Draft Report Provided to the Office of Disability, Aging, and Long‐Term Care Policy.

[hec4949-bib-0025] Congressional Budget Office . 2013. Rising Demand for Long‐Term Services and Supports for Elderly People. Congress of The United States.

[hec4949-bib-0026] Costa‐Font, J. 2010. “Family Ties and the Crowding Out of Long‐Term Care Insurance.” Oxford Review of Economic Policy 26, no. 4: 691–712. 10.1093/oxrep/grq040.

[hec4949-bib-0027] Costa‐Font, J. , and C. Courbage . 2015. “Crowding Out of Long‐Term Care Insurance: Evidence From European Expectations Data.” Health Economics 24, no. S1: 74–88. 10.1002/hec.3148. 25760584

[hec4949-bib-0028] Courtemanche, C. , and D. He . 2009. “Tax Incentives and the Decision to Purchase Long‐Term Care Insurance.” Journal of Public Economics 93, no. 1‐2: 296–310. 10.1016/j.jpubeco.2008.05.007.

[hec4949-bib-0029] Cutler, D. 1996. Why Don’t Markets Insure Long‐Term Risk? Harvard University. National Bureau of Economic Research.

[hec4949-bib-0030] De Chaisemartin, C. , and X. d'Haultfoeuille . 2020. “Two‐way Fixed Effects Estimators With Heterogeneous Treatment Effects.” American Economic Review 110, no. 9: 2964–2996. 10.1257/aer.20181169.

[hec4949-bib-0031] Deshpande, M. , and Y. Li . 2019. “Who Is Screened Out? Application Costs and the Targeting of Disability Programs.” American Economic Journal: Economic Policy 11, no. 4: 213–248. 10.1257/pol.20180076.

[hec4949-bib-0032] Eggleston, K. N. , and M. Anita . 2019. “Financing Longevity: The Economics of Pensions, Health, and Long‐Term Care: Introduction to the Special Issue.” Journal of the Economics of Ageing 13, no. May: 1–6. 10.1016/j.jeoa.2018.10.001.

[hec4949-bib-0033] Eggleston, K. N. , and V. R. Fuchs . 2012. “The New Demographic Transition: Most Gains in Life Expectancy Now Realized Late in Life.” Journal of Economic Perspectives 26, no. 3: 137–156. 10.1257/jep.26.3.137. 25076810 PMC4112481

[hec4949-bib-0035] Finkelstein, A. , and K. McGarry . 2006. “Multiple Dimensions of Private Information: Evidence From the Long‐Term Care Insurance Market.” American Economic Review 96, no. 4: 938Â–958. 10.1257/aer.96.4.938. PMC3022330 21253439

[hec4949-bib-0036] Fisher, G. G. , and L. H. Ryan . 2018. “Overview of the Health and Retirement Study and Introduction to the Special Issue.” Work Aging Retire 4, no. 1: 1–9. 10.1093/workar/wax032. 29423243 PMC5798643

[hec4949-bib-0037] Frank, R. G. 2012. “Long‐Term Care Financing in the United States: Sources and Institutions.” Applied Economic Perspectives and Policy 34, no. 2: 333–345. 10.1093/aepp/pps016.

[hec4949-bib-0038] Goda, G. S. 2011. “The Impact of State Tax Subsidies for Private Long‐Term Care Insurance on Coverage and Medicaid Expenditures.” Journal of Public Economics 95, no. 7–8: 744–757. 10.1016/j.jpubeco.2010.11.001.

[hec4949-bib-0039] Goda, G. S. , E. Golberstein , and D. C. Grabowski . 2011. “Income and the Utilization of Long‐Term Care Services: Evidence From the Social Security Benefit Notch.” Journal of Health Economics 30, no. 4: 719–729. 10.1016/j.jhealeco.2011.04.001. 21641063

[hec4949-bib-0040] Goodman‐Bacon, A. 2021. “Difference‐in‐Differences With Variation in Treatment Timing.” Journal of Econometrics 225, no. 2: 254–277. 10.1016/j.jeconom.2021.03.014.

[hec4949-bib-0041] Government Accountability Office . 2007. Long‐Term Care Insurance: Partnership Programs Include Benefits That Protect Policyholders and Are Unlikely to Result in Medicaid Savings, Report # GAO‐07‐231.

[hec4949-bib-0044] Kaiser Family Foundation . 2019. Medicaid and Long‐Term Care Quiz. https://www.kff.org/quiz/medicaid‐and‐long‐term‐care‐quiz/.

[hec4949-bib-0045] Kapteyn, A. , Michaud, P. C. , Smith, J. P. and Van Soest, A. 2006. Effects of Attrition and Non‐Response in the Health and Retirement Study.

[hec4949-bib-0046] Kemper, P. , H. L. Komisar , and L. Alecxih . 2005. “Long‐Term Care Over an Uncertain Future: What Can Current Retirees Expect?” INQUIRY: The Journal of Health Care Organization, Provision, and Financing 42, no. 4: 335–350. 10.5034/inquiryjrnl_42.4.335. 16568927

[hec4949-bib-0047] Konetza, R. 2014. Encyclopedia of Health Economics ‐ Long‐Term Care Insurance (No. ISBN 978‐0‐12‐375678‐7)

[hec4949-bib-0048] Lin, H. , and P. Jeffrey . 2013. “The Impact of the Partnership Long‐Term Care Insurance Program on Private Coverage.” Journal of Health Economics 32, no. 6: 1205–1213. 10.1016/j.jhealeco.2013.09.010. 24189449

[hec4949-bib-0049] Meiners, M. R. 2009. “Long‐Term Care Insurance Partnership: Considerations for Cost‐Effectiveness.” In Center for Health Care Strategies, Inc. Policy Brief.

[hec4949-bib-0050] Meiners, M. R. , and S. C. Goss . 1994. “Passing the ‘Laugh Test’ for Long‐Term Care Insurance Partnerships.” Health Affairs 13, no. 5: 225–228. 10.1377/hlthaff.13.5.225. 7868027

[hec4949-bib-0051] Meiners, M. R. , H. L. McKay , and K. J. Mahoney . 2002. “Partnership Insurance: An Innovation to Meet Long‐Term Care Financing Needs in an Era of Federal Minimalism.” Journal of Aging & Social Policy 14, no. 3–4: 75–93. 10.1300/J031v14n03_05. 17432478

[hec4949-bib-0052] Michaud, P. C. , A. Kapteyn , J. P. Smith , and A. Van Soest . 2011. “Temporary and Permanent Unit Non‐Response in Follow‐Up Interviews of the Health and Retirement Study.” Longitudinal and Life Course Studies 2, no. 2: 145–169.

[hec4949-bib-0053] Mommaerts, C. D. 2024. “Long‐Term Care Insurance and the Family.” Journal of Political Economy 133, no. 1: 1–52. 10.1086/732887.

[hec4949-bib-0054] National Institute on Aging and The Social Security Administration . 2018. Health and Retirement Study (U.S). http://hrsonline.isr.umich.edu/.

[hec4949-bib-0055] Nichols, A. L. , and R. J. Zeckhauser . 1982. “Targeting Transfers through Restrictions on Recipients.” American Economic Review 72, no. 2: 372–377.

[hec4949-bib-0056] Norton, E. C. 2016. “Health and Long‐Term Care.” In Handbook of the Economics of Population Aging, 951–989. Elsevier. 10.1016/bs.hespa.2016.06.001.

[hec4949-bib-0057] Norton, E. C. , and F. Sloan (1997). “Adverse Selection, Bequests, Crowding Out, and Private Demand for Insurance: Evidence From the Long‐Term Care Insurance Market.” Journal of Risk and Uncertainty 15, no. 1: 201–219. 10.1023/A:1007749008635.

[hec4949-bib-0058] NYSPLTC . 2011. New York State Partnership for Long‐Term Care. Quaterly Update 2nd Quarter. New York State Department of Health.

[hec4949-bib-0071] Oster, E. , I. Shoulson , K. Quaid , and E. R. Dorsey . 2010. “Genetic Adverse Selection: Evidence From Long‐term Care Insurance and Huntington Disease.” Journal of Public Economics 94, no. 11‐12: 1041–1050.

[hec4949-bib-0059] Pan, F. 2009. Three Essays on Health Economics (Order No. 3354044). ProQuest Dissertations & Theses Global; ProQuest One Business. https://www.proquest.com/dissertations‐theses/three‐essays‐on‐health‐economics/docview/304940694/se‐2.

[hec4949-bib-0060] Pauly, M. V. 1990. “The Rational Nonpurchase of Long‐Term‐Care Insurance.” Journal of Political Economy 98, no. 1: 153–168. 10.1086/261673.

[hec4949-bib-0061] Puhani, P. A. 2012. “The Treatment Effect, the Cross Difference, and the Interaction Term in Nonlinear ‘Difference‐In‐Differences’ Models.” Economics Letters 115, no. 1: 85–87. 10.1016/j.econlet.2011.11.025.

[hec4949-bib-0062] Reaves, E. , and M.B. Musumeci . 2015. Medicaid and Long‐Term Services and Supports: A Primer. Kaiser Family Foundation. http://files.kff.org/attachment/report‐medicaid‐and‐long‐term‐services‐and‐supports‐a‐primer.

[hec4949-bib-0063] Robert Wood Johnson Foundation (RWJF) . 2007. “Program to Promote Long‐Term Care Insurance for the Elderly.” Health Policy Snapshot.

[hec4949-bib-0064] Rothstein, J. 2007. Long‐Term Care Partnership Expansion: A New Opportunity for States. Issue Brief. Robert Wood Johnson Foundation.

[hec4949-bib-0065] Sommers, B. D. , M. Roberts Tomasi , K. Swartz , and A. M. Epstein . 2012. “Reasons for the Wide Variation in Medicaid Participation Rates Among States Hold Lessons for Coverage Expansion in 2014.” Health Affairs 31, no. 5: 909–919. 10.1377/hlthaff.2011.0977. 22566429

[hec4949-bib-0066] Sonnega, A. , J. D. Faul , M. B. Ofstedal , K. M. Langa , J. W. Phillips , and D. R. Weir . 2014. “Cohort Profile: The Health and Retirement Study (HRS).” International Journal of Epidemiology 43, no. 2: 576–585. 10.1093/ije/dyu067. 24671021 PMC3997380

[hec4949-bib-0067] Sun, L. , and S. Abraham . 2020. “Estimating Dynamic Treatment Effects in Event Studies With Heterogeneous Treatment Effects.” Journal of Econometrics 225, no. 2: 175–199. 10.1016/j.jeconom.2020.09.006.

[hec4949-bib-0068] Sun, W. , and A. Webb . 2024. “Can Long‐Term Care Partnership Programmes Increase Insurance Coverage and Reduce Medicaid Costs?” Applied Economics 56, no. 52: 6532–6546. 10.1080/00036846.2023.2274309.

[hec4949-bib-0069] Thach, N. , and J. Wiener . 2018. “An Overview of Long‐Term Services and Supports and Medicaid: Final Report.” In Office of the Assistant Secretary for Planning and Evaluation. U.S. Department of Health and Human Services. https://aspe.hhs.gov/basic‐report/overview‐long‐term‐services‐and‐supports‐and‐medicaid‐final‐report.

[hec4949-bib-0070] Zeckhauser, R. 2021. “Strategic Sorting: The Role of Ordeals in Health Care.” Economics and Philosophy 37, no. 1: 64–81. 10.1017/s0266267120000139.

