# Supplementary material for: Long‐Term Care Partnership Effects on Medicaid and Private Insurance
Source: Health Econ. 2025 Mar 15;34(6):1171–87. doi: 10.1002/hec.4949 (PMC12045773; doi:10.1002/hec.4949)
Supplement: Supplementary file 1 — Supporting Information S1 [file HEC-34-1171-s001.docx]

**Online Appendix of the paper ‘Long-Term Care Partnership Effects on Medicaid and Private Insurance’**

**Section I**

**Figure I: Coverage of private long-term care insurance (LTCI) over time.**

Note: Trends in the mean coverage of LTCI ownership for the US. (Health and Retirement Study, Wave 3-14, 1996-2018.)

**Figure II: Medicaid take-up over time.**

Note: Trends in the mean coverage of Medicaid take-up for the US. (Health and Retirement Study, Wave 3-14, 1996-2018.)

**Figure III: LTCI Individual Market Sales: 1990-2014 (Thousands)**

Source: Nordman et at. (2016), contributed for National Association of Insurance Commissioners and The Center for Insurance Policy and Research.

**Table I: Adoption of Long-Term Care Partnership Insurance Across States**

| **What States Have Approved Long-Term Care Partnership Insurance for Sale (Updated: April 2017)** | | |
| --- | --- | --- |
| **State** | **Effective Date (As of April 2017)** | **Policy Reciprocity** |
| Alabama | 03/02/09 | Yes |
| Alaska | Not Filed | --- |
| Arizona | 07/01/08 | Yes |
| Arkansas | 07/01/08 | Yes |
| California | Original Partnership | No |
| Colorado | 01/02/08 | Yes |
| Connecticut | Original Partnership | Yes |
| Delaware | 11/02/11 | Yes |
| District of Columbia | Not Filed | --- |
| Florida | 01/01/07 | Yes |
| Georgia | 01/01/07 | Yes |
| Hawaii | Pending | --- |
| Idaho | 11/02/06 | Yes |
| Illinois | Pending | --- |
| Indiana | Original Partnership | Yes |
| Iowa | 01/01/10 | Yes |
| Kansas | 04/01/07 | Yes |
| Kentucky | 06/16/08 | Yes |
| Louisiana | 10/01/09 | Yes |
| Maine | 07/01/09 | Yes |
| Maryland | 01/01/09 | Yes |
| Massachusetts | Proposed | --- |
| Michigan | Work stopped | --- |
| Minnesota | 07/02/06 | Yes |
| Mississippi | Not Filed | --- |
| Missouri | 08/01/08 | Yes |
| Montana | 07/01/09 | Yes |
| Nebraska | 07/01/06 | Yes |
| Nevada | 01/01/07 | Yes |
| New Hampshire | 02/16/10 | Yes |
| New Jersey | 07/01/08 | Yes |
| New Mexico | Not Filed | --- |
| New York | Original Partnership | Yes |
| North Carolina | 03/07/11 | Yes |
| North Dakota | 01/01/07 | Yes |
| Ohio | 09/10/07 | Yes |
| Oklahoma | 07/01/08 | Yes |
| Oregon | 01/01/08 | Yes |
| Pennsylvania | 09/15/07 | Yes |
| Rhode Island | 07/01/08 | Yes |
| South Carolina | 01/01/09 | Yes |
| South Dakota | 07/01/07 | Yes |
| Tennessee | 10/01/08 | Yes |
| Texas | 03/01/08 | Yes |
| Utah | Not Filed | --- |
| Vermont | Not Filed | --- |
| Virginia | 09/01/07 | Yes |
| Washington | 01/01/12 | Yes |
| West Virginia | 01/17/2011 | Yes |
| Wisconsin | 01/01/09 | Yes |
| Wyoming | 06/29/09 | Yes |

Source: American Association of Long-Term Care Insurance website, which comes under U.S. Government Accountability Office’s Consumer Information Center. <http://www.aaltci.org/long-term-care-insurance/learning-center/long-term-care-insurance-partnership-plans.php>)

**Table II: Baseline Models – Impact of Partnership on LTCI and Medicaid**

|  | (1) | (2) |
| --- | --- | --- |
| VARIABLES | RLTCI | Medicaid |
| Partnership (LTCIP) | 0.0154*** | -0.0082** |
|  | (0.0048) | (0.0036) |
| ACA Medicaid Expansion | -0.0041 | 0.03*** |
|  | (0.0054) | (0.0043) |
| Perm. Partnership | -0.0013 | 0.054*** |
|  | (0.022) | (0.012) |
| age | 0.003 | 0.0017 |
|  | (0.0035) | (0.0026) |
| Male | -0.0127*** | -0.01*** |
|  | (0.004) | (0.0025) |
| College_edu | 0.065*** | -0.048*** |
|  | (0.0039) | (0.0027) |
| Married | 0.023*** | -0.074*** |
|  | (0.004) | (0.00315) |
| Income | 3.8e-08** | -2.05e-08** |
|  | (1.86e-08) | (7.91e-09) |
| White | 0.0175*** | -0.082*** |
|  | (0.0038) | (0.005) |
| Fair/Poor Health | -0.029*** | 0.079*** |
|  | (0.0033) | (0.0036) |
| Cancer | 0.0097 | -0.0011 |
|  | (0.0064) | (0.0043) |
| Arthritis | 0.0039 | 0.012*** |
|  | (0.004) | (0.0024) |
| Diabetes | -0.0072 | 0.0187*** |
|  | (0.0045) | (0.004) |
| Strokes | -0.0126* | 0.048*** |
|  | (0.0067) | (0.0086) |
| Heart Disease | 0.0024 | 0.022*** |
|  | (0.005) | (0.0041) |
| Psycho. Disease | -0.0105** | 0.05*** |
|  | (0.0053) | (0.005) |
| Lung Disease | -0.012** | 0.052*** |
|  | (0.0053) | (0.005) |
| Constant | -0.14 | 0.108 |
|  | (0.11) | (0.084) |
| STATE + YEAR FE | YES | YES |
| Observations | 156,102 | 156,102 |
| R-squared | 0.035 | 0.142 |

*significant at 10%; ** significant at 5%; *** significant at 1%, robust standard error clustered at state and household level. All the coefficient estimates are weighted using survey weights at person-level.

Note: The estimates are obtained using the sample from Health and Retirement Study, Waves 3-14, 1996-2018. Each coefficient indicates OLS estimates of equation (1). There are two dependent variables namely Private long-term care insurance (LTCI) and public long-term care insurance or Medicaid. The variable ‘Partnership’ is a treatment variable, which is a binary indicator for whether the state adopted Partnership program in a given year after the passage of Deficit Reduction Act (DRA-2005). It is also called as ‘Partnership’ or ‘LTCIP’. At first, we estimate the impact of Partnership on LTCI in which Column (1) includes Medicaid expansion reform, a set of demographic controls, and chronic diseases such as cancer, diabetes, stroke, heart disease, lung disease, psychological disease, and arthritis. Whereas Columns (2) follows the similar procedure for Medicaid.

**Section II**

*Placebo Test.* We run our main model using several unrelated dependent variables to make sure that the estimated effect of partnership is not driven by pension, employer health insurance, and work for pay. Table III reports evidence of statistically non-significant effects, consistent with the expected estimates of a placebo test.

**Table III: Placebo tests**

|  | Pension | Work for pay | Employer Health insurance |
| --- | --- | --- | --- |
|  | (1) | (2) | (5) |
|  |  |  |  |
| **Partnership** | 25.72 | 0.0062 | 0.0034 |
|  | (213.5) | (0.007) | (0.0076) |
|  |  |  |  |
|  |  |  |  |
| **State + Year Fixed Effects** | YES | YES | YES |
| **Set of Controls** | YES | YES | YES |
| **N** | 148,061 | 148,000 | 146,629 |

Note: All the coefficient estimates are weighted using survey weights at person-level. The estimates are obtained using the sample from Health and Retirement Study, Waves 3-14, 1996-2018, run as placebo tests on other outcomes (Pension, work for pay, and employer health insurance). Each coefficient indicates OLS estimates of equation (2). All models are inclusive of state as well as year fixed effects and control variables (demographic controls, ACA-Medicaid, and a set of chronic diseases).

**Table IV – Pre-trend test: Impact of LTCIP on LTCI Ownership and Medicaid Uptake**

|  | LTCI Ownership | Medicaid Uptake |
| --- | --- | --- |
| Year98*Partnership | -0.0013 | -0.0034 |
|  | (0.0092) | (0.0052) |
| Year00* Partnership | -0.0043 | -0.007 |
|  | (0.0095) | (0.0057) |
| Year02*Partnership | 0.007 | -0.0088 |
|  | (0.01) | (0.006) |
| Year04* Partnership | 0.0072 | -0.006 |
|  | (0.01) | (0.0062) |
| p-value of the F test for the null: Coefficients are jointly 0 | 0.56 | 0.6 |

Data restricted to years 1996-2004, omitted year is 1996, the outcome are LTCI ownership and Medicaid uptake. We interact year effects with the partnership states, conditioning on state and year fixed effects, and we test the null hypothesis that the interaction of year dummies with the partnership states are jointly 0.

**Section III**

**Welfare Analysis: A MVPF Approach**

We attempt to evaluate the welfare impact of partnership. Therefore, to analyse the partnership insurance policy, we use Marginal Value of Public Funds (MVPF hereafter) approach suggested by (Hendren 2013; Finkelstein and Hendren 2020; Hendren and Sprung-Keyser 2020). The MVPF is an elegant way of linking causal estimates of a policy to the welfare analysis of that policy. As per Hendren (2016) and Finkelstein and Hendren (2020), the MVPF is defined as the ratio of marginal benefits to the marginal cost of the policy, as shown in equation I.

$$MVPF=\frac{"Benefits"}{"Costs"} (I)$$

The numerator refers to the benefit received by a recipient after a policy change. This is equivalent to the willingness to pay for the increased expenditure due to policy (Finkelstein and Hendren 2020; Hendren and Sprung-Keyser 2020). The denominator reflects the costs to the government for the implementation of a policy. It consists of two categories of costs viz. Mechanical Cost and Fiscal Externality of the policy. The mechanical cost of the policy refers to increase in government expenditure post-adoption of partnership unaccompanied by any behavioural response. In the context of partnership, we assume that the mechanical cost is either zero or miniscule, because the direct cost of the partnership program consists of administrative costs of making the policy available for purchase. Such costs are miniscule as a recipient can choose an option of partnership in place of regular insurance policy while buying a contract from the same provider. Therefore, we continue to assume the mechanical cost of partnership as zero or miniscule.

Let ‘X’ be the additional coverage purchased by an individual, then Equation (II) indicates the marginal value of public funds (MVPF) (Hendren 2013; Finkelstein and Hendren 2020; Hendren and Sprung-Keyser 2020) when MM group individual with fewer resources over insuring their assets. Where A, C, & P indicate the protected assets, insurance coverage, and premium in $ respectively; for denominator, let M and t indicate Medicaid costs and tax on earnings, in $ amount.

$$MVPF= \frac{A+C-P}{(M\pm t-X)} (II)$$

These negative costs to the government (or Medicaid savings) also signify that the government spending pays for itself and MVPF is defined as infinite (Hendren and Sprung-Keyser, 2020). Overall, the partnership improves the welfare of an MM groups and at the same time reduces a cost of providing Medicaid.

The fiscal externality (FE) refers to costs incurred due to the behavioural response after the adoption of policy. In case of partnership, the behavioural response can occur through A) Decrease/increase in labour participation after the adoption of partnership. Decrease in labour participation means that an individual does not need to accumulate money to finance their future long-term care costs once they are covered and their assets are protected through partnership. This leads to decrease in income tax revenue collected by government, a negative fiscal externality. However, an increase in labour participation means that individual may intend to accumulate money to satisfy other motives including transfer of bequest which in turn increases the income tax revenue collected by the government and results in a positive fiscal externality for the government. Our estimates indicate that partnership increases the labour participation for elderly, but they are not significant. B) Another behavioural response of the policy can result in increase in government expenditure (or decrease in costs) if an individual happens to purchase more coverage than the assets she intends to protect. Such an additional coverage may ultimately result in decrease in Medicaid costs to government, a positive fiscal externality for government. Hence, we infer that the Medicaid savings we find in our simulation analysis comes from such a behavioural response to the policy. Equation (III) includes the various components of benefits and costs after partnership adoption. For numerator, let A, C, & P indicate the protected assets, insurance coverage, and premium in $ respectively; for denominator, let M, t, & X indicate Medicaid costs, tax on earnings, and additional coverage in $ respectively.^[[1]](#footnote-1)^

$$MVPF=\frac{"Benefits"}{MC+FE}= \frac{A+C-P}{\left( 0 \right)+(M\pm t-X)} (III)$$

For simplicity, we take an example of a median wealth individual with a wealth of $144,000 for our analysis and observe that the welfare analysis of partnership results in three different scenarios depending upon how a marginal beneficiary behaviourally responds to the adoption of partnership (National Institute on Aging and The Social Security Administration 2018). We continue to assume that a policy can be purchased at an annual premium of θ = $2,000 and that private LTCI coverage provides a daily benefit of $100 for a 65-year-old individual. We observed that, in the absence of partnership, a median wealth individual needed to spend down her assets to $2000 before qualifying for Medicaid. Thus, a median wealth individual required to spend $142,000 of her assets, after the exhaustion of private insurance coverage, before becoming eligible for a public insurance via Medicaid. The MVPF associated with no- partnership is shown in row 1 of Table VI.

However, in the presence of partnership, an individual is provided with an option of discounting her assets before qualifying for Medicaid. In an optimal scenario, a median wealth individual can protect all of her assets by purchasing partnership policy with a private coverage equivalent to her assets ($144,000 - $2000 = $142,000). It is important to notice that the exact optimal planning via partnership does not affect the Medicaid expenditure and Medicaid costs remains same with or without partnership. Nevertheless, it can be observed that the benefits received by an individual with partnership policy increase by an amount of assets she protects under the provision of partnership. For an individual with private insurance coverage and keeping other things constant, we find that MVPF of partnership (row 2 of Table VI) is greater than MVPF without partnership (row 1). Thus, we can infer that partnership improves the welfare of an individual.

Additionally, if a median wealth individual purchases insurance through partnership with a coverage less than her total assets (<$142,000), then she pays the difference between amount of coverage and Medicaid threshold out of her own pocket before qualifying for Medicaid.^[[2]](#footnote-2)^ Let that difference be represented by ‘d’. But once again it is important to note that this will not change the government expenditure of providing public insurance via Medicaid (ref. row 3 of Table VI).

Finally, given that the insurance premium varies by gender, age, health conditions, benefit multiplier, and couple status, and comes in several standardized packages. Therefore, buying an optimal coverage becomes a rare possibility, and an individual may end up purchasing a coverage greater than her assets. However, this additional coverage has a direct impact on the Medicaid costs; it leads savings in Medicaid and reduce the fiscal burden on the government. Let ‘X’ be the additional coverage purchased by an individual, row 4 of Table VI indicate the MVPF with coverage above optimal level. We find that MVPF associated with row 4 of Table VI will be greater than previous cases. These negative costs to the government also signify that the government spending pays for itself and MVPF is defined as infinite (Hendren and Sprung-Keyser 2020). Overall, the partnership improves the welfare of an individual without raising the costs to the government for providing Medicaid.

**Table V: Welfare Analysis using MVPF Approach.**

| **Sr No** | **Scenarios** | **Coverage** | **MVPF** |
| --- | --- | --- | --- |
| 1) | No-Partnership | ------ | $= \frac{C-P-A}{(M\pm t)}$ |
| 2) | Partnership – Optimal | C = $142,000 | $= \frac{A+C-P}{(M\pm t)}$ |
| 3) | Partnership – Below Optimal | C < $142,000 | $= \frac{A+C-P-d}{(M\pm t)}$ |
| 4) | Partnership – Above Optimal | C > $142,000 | $= \frac{A+C-P}{(M\pm t-X)}$ |

Note: This table consists of four different scenarios and their corresponding marginal values of public funds (MVPF) respectively. The coverage estimates, indicative of average individual wealth, comes from the Health and Retirement study (1996-2018).

**Table VI: Variable Description**

| Variables | Definition |
| --- | --- |
| Dependent Variables | |
| *LTCI* | Equals 1 if respondent purchased LTCI, else 0. |
| *Medicaid* | Equals 1 if respondent is on Medicaid, else 0. |
| Treatment | |
| *Partnership* | Equals 1 if state adopted LTC Partnership program, else 0. |
| Control Variables | |
| *Married* | Equals 1 if respondent is married, else 0. |
| *Income* | Total household income. |
| *Male* | Equals 1 if respondent is Male, else 0. |
| *Child* | Equals 1 if respondent has any children, else 0. |
| *Age* | Age of a respondent. |
| *College* | Equals 1 if respondent has college education or more, else 0. |
| *White* | Equals 1 if respondent is white American, else 0. |
| *Fair/Poor Health* | Equals 1 if respondent has fair or poor health, else 0. |
| *Cancer* | Equals 1 if respondent has a cancer disease, else 0. |
| *Arthritis* | Equals 1 if respondent has arthritis disease, else 0. |
| *Diabetes* | Equals 1 if respondent has diabetes, else 0. |
| *Stroke* | Equals 1 if respondent had a stroke, else 0. |
| *Heart Disease* | Equals 1 if respondent has a heart disease, else 0. |
| *Psycho. Disease* | Equals 1 if respondent has a psychological disease, else 0. |
| *Lung Disease* | Equals 1 if respondent has a lung disease, else 0. |
| *Permanent Partnership* | Equals 1 if a state belongs to permanent partnership (CA, CT, IN, NY), else 0. |

**Table VII: List of States Participated in LTC-state tax subsidy.**

| **State** | **Effective Year** | **Policy Reciprocity** |
| --- | --- | --- |
| Alabama | 1995/96 | Yes |
| Alaska | -- | No |
| Arizona | -- | No |
| Arkansas | 2007/08 | Yes |
| California | 2007/08 | Yes |
| Colorado | 1999/00 | Yes |
| Connecticut | -- | No |
| Delaware | -- | No |
| District of Columbia | 2005/06 | Yes |
| Florida | -- | No |
| Georgia | -- | No |
| Hawaii | 2007/08 | Yes |
| Idaho | 2001/02 | Yes |
| Illinois | -- | No |
| Indiana | 1999/00 | Yes |
| Iowa | 1997/98 | Yes |
| Kansas | 2005/06 | Yes |
| Kentucky | 1997/98 | Yes |
| Louisiana | 2007/08 | Yes |
| Maine | 1992 | Yes |
| Maryland | 1999/00 | Yes |
| Massachusetts | -- | No |
| Michigan | -- | No |
| Minnesota | 1999/00 | Yes |
| Mississippi | 2007/08 | Yes |
| Missouri | 1999/00 | Yes |
| Montana | 1992 | Yes |
| Nebraska | 2005/06 | Yes |
| Nevada | -- | No |
| New Hampshire | -- | No |
| New Jersey | 2007/08 | Yes |
| New Mexico | 1999/00 | Yes |
| New York | 1995/96 | Yes |
| North Carolina | 1999/00 | Yes |
| North Dakota | 1994 | Yes |
| Ohio | 1999/00 | Yes |
| Oklahoma | -- | No |
| Oregon | 1999/00 | Yes |
| Pennsylvania | -- | No |
| Rhode Island | -- | No |
| South Carolina | -- | No |
| South Dakota | -- | No |
| Tennessee | -- | No |
| Texas | -- | No |
| Utah | 1999/00 | Yes |
| Vermont | -- | No |
| Virginia | 1999/00 | Yes |
| Washington | -- | No |
| West Virginia | 1999/00 | Yes |
| Wisconsin | 1997/98 | Yes |
| Wyoming | -- | No |

**References:**

Finkelstein, A., and K. McGarry. 2006. “Welfare Analysis Meets Causal Inference.” *Journal of Economic Perspectives* 34, no. 4: 146 – 167. <https://doi.org/10.1257/jep.34.4.146>

Hendren, N. 2013. *The Policy Elasticity.* Cambridge: National Bureau of Economic Research. <https://doi.org/10.3386/w19177.w19177>

Hendren, N., and B. Sprung-Keyser. 2020. “A Unified Welfare Analysis of Government Policies.” *Quarterly Journal of Economics* 135, no. 3: 1209-1318.

1. It is difficult to distinguish between a policy purchased through partnership and using tax-subsidy, but our estimates are robust to the inclusion of tax subsidy in the model. It is not straightforward to calculate the cost of implementation of partnership but given that the partnership policy can be purchased through the same exchanges we can assume that the adoption of partnership incurred minimal or no cost to the government. It is also difficult to identify the costs imposed on the government via Medicaid by an individual holding partnership policy and getting qualified for Medicaid after exhausting her coverage. Therefore, our welfare analysis of partnership does not include the exact cost of Medicaid in the MVPF formula. [↑](#footnote-ref-1)
2. For example, if she buys a coverage of $100,000, then the difference she needs to pay out of her pocket would be $42,000. Overall, it is not optimal for a median individual to purchase coverage less than $142,000. [↑](#footnote-ref-2)
